# ZIF-71-Coated CuO:Al with Enhanced Gas-Sensing Performance for *n*‑Butanol and Hydrogen

**DOI:** 10.1021/acsaelm.5c01659

**Published:** 2025-11-12

**Authors:** Rajat Nagpal, Masaya Sugihara, Cristian Lupan, Tim Tjardts, Nahomy Meling-Lizarde, Thomas Strunskus, Haoyi Qiu, Rainer Adelung, Rob Ameloot, Oleg Lupan

**Affiliations:** † Chair for Functional Nanomaterials, Department of Materials Science, Faculty of Engineering, 9179 Kiel University, Kaiserstraße 2, Kiel D-24143, Germany; ‡ Center for Nanotechnology and Nanosensors, Department of Microelectronics and Biomedical Engineering, Faculty CIM, 187225 Technical University of Moldova, 168 Stefan Cel Mare str., Chisinau MD-2004, Republic of Moldova; § Centre for Membrane Separations, Adsorption, Catalysis, and Spectroscopy, 26657 KU Leuven, Leuven 3001, Belgium; ∥ Chair for Composite Materials, Department of Materials Science, Faculty of Engineering, 529440 Kiel University, Kaiserstraße 2, Kiel D-24143, Germany; ⊥ Kiel Nano, Surface and Interface Science KiNSIS, Kiel University, Christian Albrechts-Platz 4, Kiel 24118, Germany

**Keywords:** ZIF-71, metal−organic-frameworks, n-butanol, polarizability, adsorption, affinity, synergistic, dipole, sensor

## Abstract

In this study, hybrid materials combine metal-organic framework (MOF)-zeolitic imidazolate framework (ZIF) with inorganic metal-oxide semiconductors, resulting in composite materials that exhibit synergistic properties and improves the sensing capabilities of organic/inorganic hybrid sensors. MOFs provide tunable porosity and structural flexibility. Therefore, they present significant potential for gas-sensing applications. In this study, we report a simple and scalable approach to fabricate MOF ZIF-71-coated Al-doped CuO-based (CuO:Al) hybrid gas sensors. CuO:Al films with triangular-shaped grains were synthesized via a chemical solution approach. Comprehensive structural and surface analyses were conducted by using XRD, SEM, and XPS to elucidate crystallinity, morphology, and chemical states. The analysis revealed a highly crystalline structure with well-distributed ZIF-71 nanoparticles exhibiting a broad particle size range (500 to 750 nm) on the CuO:Al film. Raman spectroscopy and nitrogen (N_2_) adsorption–desorption isotherms were employed to assess the vibrational properties and porosity. These techniques provided local bonding information on the linker arms and demonstrated the microporosity and pore size distribution of the ZIF-71 nanoparticles. Thermal stability was evaluated by thermogravimetric analysis, confirming the sensor’s robustness under operational conditions. Gas-sensing studies reveal that the ZIF-71-coated CuO:Al sensors exhibit enhanced response (*S* = 11%) toward *n*-butanol at 200 °C and hydrogen gas (*S* = 61%) at an operating temperature of 250 °C. Due to *n*-butanol’s strong affinity for ZIF-71 and its high interaction strength at 200 °C, the resulting adsorption capacity improves detection sensitivity at the ZIF-71/CuO:Al interface in the developed hybrid sensor. Good repeatability and durability of the fabricated gas sensors were observed for hydrogen gas in humid conditions. The gas-sensing performance was measured at 21-day intervals. During each measurement, the sample was exposed to elevated temperatures, effectively subjecting it to thermal treatment. As a result, improved sensing performance was observed after each measurement, which was attributed to the cumulative effects of thermal exposure. These findings highlight the potential of the MOF-ZIF-71-coated CuO:Al hybrid sensors for selective, humidity-tolerant, and stable detection of *n*-butanol and hydrogen gas. Further optimization is suggested to enhance the selectivity and lower the operational temperature.

## Graphical abstract

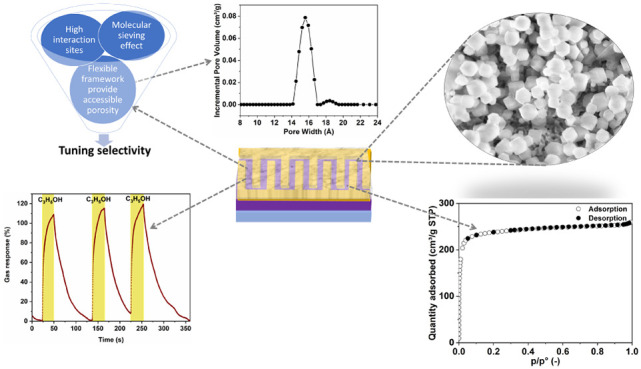

## 1 Introduction

The continuous monitoring of volatile organic compounds (VOCs) and various other gaseous species such as hydrogen is crucial in modern applications. Among VOCs, *n*-butanol is particularly relevant because prolonged exposure to *n*-butanol may cause health issues, including damage to the central nervous system and skin irritation.^1^ Consequently, many countries have established concentration limits for *n*-butanol in various environments. For instance, the permissible exposure limit is 65.9 ppm in China and 100.2 ppm in the United States, as defined by the Occupational Safety and Health Administration (OSHA).^2^ These limits are specified under standard atmospheric pressure and room temperature conditions.

Hydrogen gas is also considered a promising alternative fuel for energy applications. However, its combination with other fuels poses hazards, such as fire or explosion.^3^ Moreover, hydrogen gas storage container materials can promote hydrogen embrittlement and facilitate hydrogen permeation;^4^ consequently, leaks during transport or storage pose significant safety hazards. Thus, monitoring hydrogen gas leaks has become increasingly important across industries to ensure quick gas leak detection and prevent accidents.

The adverse effect of humidity on gas-sensing properties of metal oxides is a well-known issue and poses a limiting factor for the commercialization and practical applications of devices based on such nanostructured materials. For example, exposure to humidity reduces the concentration of ionosorbed oxygen, thereby lowering the response.^5^

Detecting hydrogen gas in high-humidity conditions is critical for energy applications.^6^ In major cities across the United States of America, outdoor humidity often exceeds 50%.^7^ Thus, hydrogen gas leak detection under real-world conditions is essential. Similarly, controlling relative humidity is crucial for *n*-butanol detection in lithium-ion battery manufacturing (≤10% during packaging, < 0.5% during manufacturing).^8^

To reduce the effect of humidity, various strategies have been explored. For example, Zn doping in copper oxide has been reported to diminish the influence of humidity on the gas-sensing response.^9^ Another method involves using hydrophobic metal–organic frameworks (MOFs), which are effective in maintaining gas-sensing performance under high relative humidity conditions.^10^

Metal oxide-based gas sensors show high sensitivity to multiple analytes and can be used for the detection of VOCs, combustible, and toxic gases for various applications.^11,12^ Copper oxide (CuO) is a common metal oxide *p*-type semiconductor that has attracted attention in the field of gas sensing. Furthermore, copper oxides are abundant and nontoxic materials that can be fabricated by suitable low-cost processes.^13^ With regard to nanoscale CuO gas sensors, several promising approaches have been reported. For example, Lupan et al. demonstrated enhanced sensing properties of a CuO/Cu_2_O mixed-phase nanocrystalline film for the detection of ethanol vapor.^14^ Additionally, Choi et al. reported a study on an Ag nanoparticle-doped CuO/Cu_2_O nanopattern-based gas sensors that exhibit good sensing performance for various VOCs at a lower detection range.^15^ Furthermore, Zhao et al. demonstrated nanopatterned CuO nanowire-based hydrogen gas sensing at 300 °C that exhibits a sensitivity of 11% for a concentration of 100 ppm.^16^ The effect of nanocomposites or doping is significant on the sensing performance of CuO. For instance, the combination of CuO with the electrically insulating aluminosilicate montmorillonite (MMT) demonstrated great enhancement (∼4 times) in sensing response to 200 ppm of *n*-butanol compared to bare CuO at 260 °C.^17^ This was attributed to changes in hole concentrations from *n*-butanol-CuO/MMT interactions at the nanocomposite junction, leading to resistance changes and improved sensing response. Despite the significance of monitoring both alcoholic series and hydrogen gas, few studies have successfully demonstrated a single sensor platform that can reliably detect both gas types. For instance, Tournier et al.^18^ reported on SnO_2_-based gas sensors that exhibit effective ethanol sensing at low temperatures (≤150 °C) and hydrogen gas sensing at high temperatures (≥250 °C). Both hydrogen gas and *n*-butanol are recognized as renewable fuels; however, *n*-butanol, due to its lower energy density, requires higher fuel consumption compared to gasoline. This challenge can be mitigated by incorporating hydrogen into *n*-butanol.^19^ Hydrogen, as a clean energy source with zero carbon emissions and a high calorific value, can help extend the combustion efficiency of engines fueled by a blend of *n*-butanol and hydrogen. The blended fuel of *n*-butanol and hydrogen is delivered through a port fuel injection system, necessitating effective gas leak detection for safety. Consequently, a highly compatible gas detector is crucial for monitoring fuel leak detection within the blended fuel supply system.^20^ The effect of Al doping has been studied in metal oxide semiconductor-based sensors. Due to the lattice mismatch between Al^3+^ (0.53 Å) and Cu^2+^ (0.73 Å) cations, defect formation can occur, potentially increasing surface area.^21^ Furthermore, Yoo et al. demonstrated the impact of Al doping in oxides on their VOC gas-sensing performance. They showed that ZnO nanoparticles doped with 1 at% Al showed a ∼4.4-fold enhancement in acetone sensing response, attributed to increased oxygen vacancy density.^22^

MOFs are a class of high-surface-area porous crystalline materials consisting of metal centers and organic ligand bridges linking the framework.^23^ They are of great interest as a general porous material due to their controlled atomic-scale features in the spatial domain, which form well-defined structures.^24^ Classical inorganic porous solids such as zeolites and hybrid porous solids such as MOFs are conceptually the same, consisting of secondary building units (SBUs). MOFs have an advantage over conventional zeolites in terms of flexibility and functionality, as their structure can be tailored by varying metal nodes and organic linkers, enabling their use in diverse applications such as gas storage,^25^ catalysis,^26^ and gas sensing.^27^ The framework structure of MOFs is not disturbed even after solvent extraction due to their weaker solvent-framework interaction.^28^ This provides accessible porosity, unlike the porosity of zeolites.

Zeolitic-imidazolate frameworks (ZIFs) are a subgroup of MOFs composed of metal centers coordinated with imidazolate linkers, typically synthesized via a self-assembly process. ZIFs possess the properties of both MOFs and zeolites, including high adsorption capacity and exceptional thermal and chemical stability.^29^ The hybrid materials integrate organic ZIFs with inorganic metal oxides to create novel materials that exhibit synergistic properties, enhancing performance across a variety of applications.^30^ Specifically, hybridization in sensors addresses the limitations of metal oxides and improves the sensing performance of organic/inorganic hybrid sensors.^31,99^ Previously,^32^ a gas sensor based on ZnO coated with ZIF-8 was reported, which exhibited good hydrogen gas selectivity over methane due to the molecular sieving effect of the ZIF-8 layer on the ZnO surface. Other MOFs, such as ZIF-71 and ZIF-7, are also used in gas-sensing applications. These materials not only exhibit molecular sieving properties but also possess a high density of interaction sites, which enhances selectivity toward analytes regardless of their molecular size.^33,34^ Zhou et al. reported^35^ the synergistic effect of ZIF-71 and WO_3_ for enhancing the H_2_S sensing response at the interface of WO_3_@ZIF-71 core–shell nanorods. This was attributed to the strong interaction between H_2_S and the organic ligands of ZIF-71. In other reported studies,^36^ ZIF-71-coated ZnO nanorod arrays showed an improved sensing response toward acetone and ethanol compared to bare ZnO nanorod arrays, resulting in a lower detection limit. MOF-coated metal oxide semiconductor gas sensors exhibit permanent microporosity and high surface area, which increases the selectivity of the target analyte by enabling preferential interaction at specific sites within the ZIF framework.^37^

MOF deposition onto metal oxide semiconductors-coated interdigitated electrodes (IDEs) is typically achieved by using controlled deposition techniques such as Langmuir–Blodgett^38^ or manual deposition techniques like drop-casting or spin-coating.^39^ *In situ* deposition, while potentially advantageous, often necessitates stringent synthesis equipment.^40^ Conversely, MOF-layer transfer techniques, including the Langmuir–Blodgett method, may induce defect formation or compromise substrate adhesion during the transfer process.^41^ Drop-casting, a simple, straightforward, yet cost-effective approach, allows for the deposition of MOFs between the IDE fingers.^42^ However, it can result in non-uniform full coverage and limited control over the spatial distribution of MOF particles.^43^ While repeated iterations to achieve the desired coverage can mitigate this, they may adversely affect conductivity.^44^

The current study aims to design chemiresistive hybrid sensors that function under various conditions by employing a controlled, simple, and cost-efficient drop-casting method to deposit metal–organic framework ZIF-71 dispersion between Au-IDEs on CuO:Al films. To the best of our knowledge, there are no prior reports on the properties or gas-sensing applications of ZIF-71-coated CuO:Al films. Sensing properties were investigated for various gases (acetone, n-butanol, methane, hydrogen, ammonia, and 2-propanol) due to their diverse properties and relevance to various applications.^32,45,46^ A sensing mechanism is proposed for its interaction with target analytes such as hydrogen or n-butanol to elucidate the role of the ZIF-71 layer. In this work, the fabricated gas sensor exhibits an approximately 110-fold enhancement in sensitivity to *n*-butanol at 200 °C upon coating a ZIF-71 layer on the CuO:Al film. The fabricated gas sensor shows good humidity tolerance and fast recovery time for the sensing of hydrogen gas. Reproducibility of gas-sensing performance was investigated after 21 days, observing an increase in gas-sensing response, while higher humidity showed that the sensing device becomes selective to hydrogen gas. Understanding the gas-sensing behavior of ZIF-71-coated CuO:Al is essential for advancing the design of selective, humidity-tolerant, and efficient chemiresistive hybrid sensors that function under various conditions.

## 2 Experimental Section

### 2.1 Materials and Sample Preparation

CuO:Al films were deposited on glass substrates (12 mm × 14 mm × 1 mm) using the synthesis from chemical solution (SCS) method, as reported previously.^14,47^ The glass substrates were cleaned using HNO_3_ (30%) solution for 10 min and then rinsed in deionized (DI) water. Thereafter, the substrates were ultrasonically cleaned in a mixture of ethanol and acetone (in equal proportions) for 5 min and then rinsed again in DI water and dried in a dry air flux. A solution of a copper thiosulfate complex was prepared as a cationic precursor, comprising 1 mol/L copper sulfate pentahydrate (99%, Ecochimie SRL) and 1 mol/L sodium thiosulfate pentahydrate (98.5%, Ecochimie SRL). The complex solution was diluted with DI water to obtain a 0.1 mol/L copper concentration under continuous stirring at room temperature. Following the dilution of the cationic precursor solution, Al doping was performed by adding 10 mg of aluminum nitrate hexahydrate (Al­(NO_3_)_3_ 9H_2_O, Alfa Aesar), previously dissolved in 100 mL of DI water, to the diluted solution.^48^ The anionic precursor used in the solution comprises 2 mol/L of sodium hydroxide (99%, Ecochimie SRL) in DI water and was continuously stirred at 80 °C. The precleaned glass substrate was immersed in the solutions using an articulated robot to avoid human error in dipping time and the number of SCS cycles.^14^ After deposition of the CuO:Al film, the sample was dried under a dry air flux. To enhance the crystallinity of the thin film, postgrowth treatment via rapid thermal annealing at 650 °C for 60 s was performed, as previously reported.^47^

To obtain a ZIF-71 dispersion, 1 mmol of zinc acetate dihydrate (99%, Sigma-Aldrich) and 2 mmol of 4,5-dichloroimidazole (97%, Tokyo Chemical Industry Co., Ltd.) were dissolved separately in 20 mL of methanol (99.9%, Fisher Chemical) each. The latter solution was rapidly added to the former solution under stirring. After 1 h of stirring, white solids were separated from the dispersion by centrifugation, followed by washing with methanol and 2-propanol (99.5%, Sigma-Aldrich). The resultant powder was kept in 2-propanol to prevent aggregation. Prior to the deposition of ZIF-71 on the CuO:Al film, Au-IDE contacts (175 nm thick) were patterned onto the CuO:Al film using a metal mask in a meander configuration, maintaining a 1 mm gap between adjacent electrodes, as previously reported.^49^ A dispersion of ZIF-71 nanoparticles was drop-cast onto the CuO:Al film using a micropipette with a 100 μL volume capacity, employing successive drop-casting steps. Initially, 50 μL of the dispersion was applied. Following a 15 min sonication period, a subsequent 50 μL aliquot, resulting in a total deposition of 100 μL, was applied. Figure 1 shows a schematic of the fabrication process of the ZIF-71-coated CuO:Al film, namely, the MOF/MOS hybrid sensor structure.

**Figure 1:**
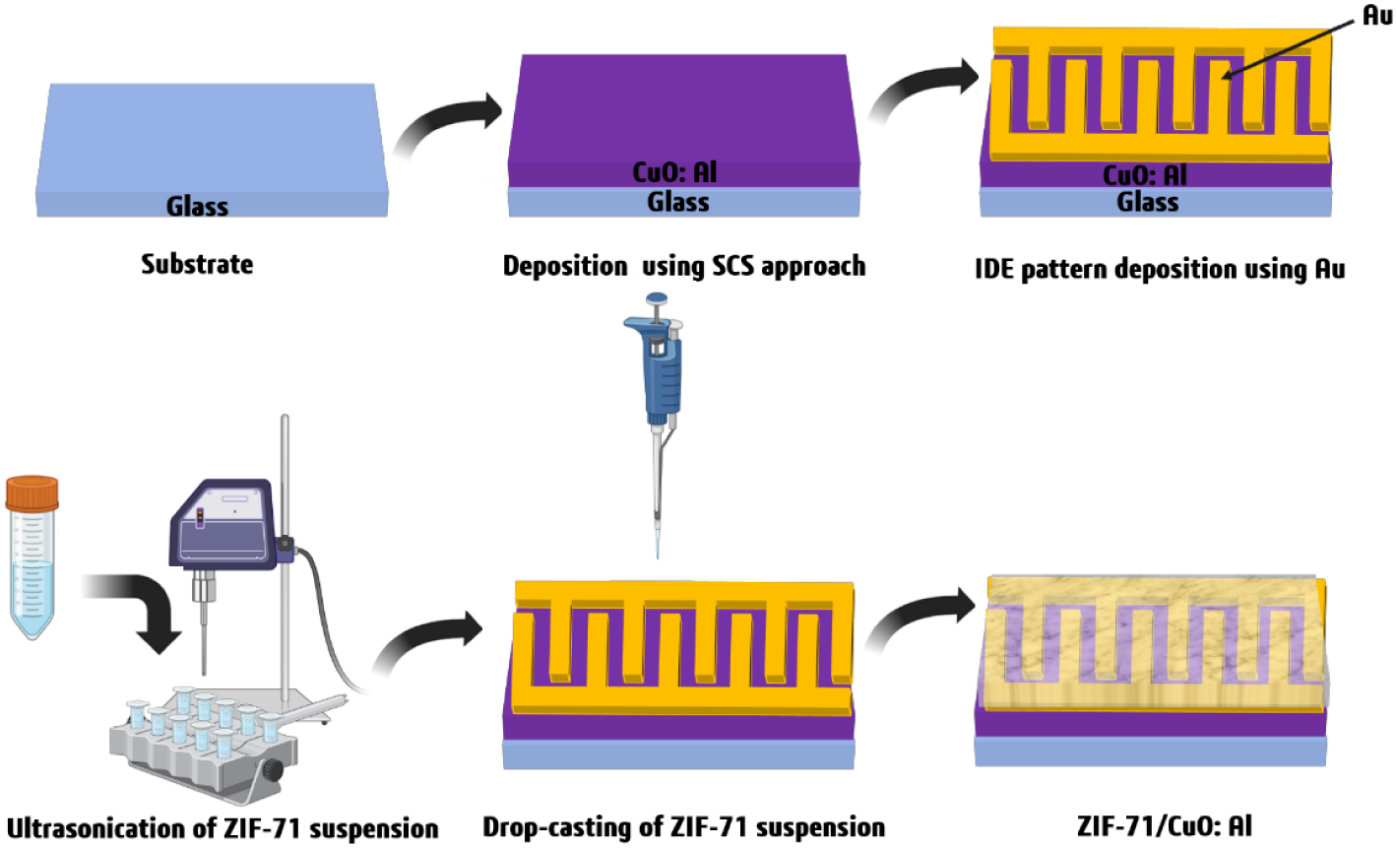
Schematic of the fabrication of the metal–organic framework ZIF-71-coated CuO:Al film-based hybrid gas sensor.

### 2.2 Characterization

XRD measurements were carried out using a Rigaku diffractometer with a high-flux X-ray source (9 kW) and Cu–Kα1 radiation (1.54 Å), operating in PB (parallel beam) configuration over a 2θ range of 2 to 100° with a step size of 0.01°. The thin-film X-ray diffractometer unit was operated at 45 kV and 200 mA to record the XRD pattern. The HyPix-3000 hybrid photon counting (HPC) one-dimensional detector mode (line mode) was used for the collection of high-resolution data.

The morphological and compositional studies of the metal–organic framework ZIF-71-coated CuO:Al samples were conducted using SEM (Zeiss, 7 kV, 10 μA) and energy-dispersive X-ray spectroscopy (EDX, Zeiss Gemini Ultra55 Plus, 15 kV), respectively.

The micro-Raman spectrometer (WITec alpha300 RA system) was used to check the intactness of the metal–organic framework ZIF-71 on the CuO:Al film based on the presence of its characteristic vibrational bands. It is equipped with a triple grating (1200 grooves/mm) system with a blaze wavelength of 500 nm and a charge-coupled device (CCD) detector for signal acquisition. The excitation source used was a 532 nm line from an Nd:YAG laser, with an optical power of 8 mW.

N_2_ physisorption on bulk ZIF-71 particles was performed at 77 K using a Micromeritics 3Flex instrument. Prior to the measurement, the sample was activated at 200 °C under vacuum for 12 h.

Thermogravimetric analysis (TGA) was conducted on an STA 449 F3 Jupiter (Netzsch, Germany) under a flow of synthetic air (50 mL/min). The samples were analyzed from 25 to 800 °C with a heating rate of 5 °C/min.

XPS measurements of the ZIF-71-coated CuO:Al films were carried out in an ultrahigh vacuum (UHV) XPS system (PREVAC spectrometer) using a nonmonochromatic Al Kα X-ray source (1486.6 eV), operated at 300 W (15 kV, 20 mA). The base pressure within the analysis chamber was maintained in the 10^–8^–10^–9^ mbar regime, achieved by using a turbomolecular pump in conjunction with a scroll backing pump. Survey spectra were recorded over a binding energy range of 0 to 1300 eV at a pass energy of 200 eV with three iterations. High-resolution spectra were recorded over a relevant binding energy range at a pass energy of 50 eV with 20 iterations. Spectral deconvolution and peak fitting were performed using CASA XPS software (version 2.3.23), following the Shirley-type background evaluation and Gaussian–Lorentzian (1:1) peak fitting models. Charge correction was applied by setting the Zn 2p_3/2_ peak to 1021.7 eV and shifting all the spectra accordingly.^50^ The chemical surface composition was determined by numerically integrating the peak areas after subtracting the Shirley background, followed by applying the corresponding relative sensitivity factors.

### 2.3 Gas-Sensing Experiments

The samples were exposed to a range of VOCs (acetone, *n*-butanol, and 2-propanol), as well as gases (hydrogen, methane, and ammonia), each at 100 ppm in air, across operating temperatures from 20 to 250 °C. The test setup consisted of a closed chamber, a gas flow system, and a source meter (Keithley 2400), controlled via a LabView interface, as previously reported.^51^

Using mass flow controllers, test gases and air were mixed to obtain a 100 ppm concentration, as previously reported.^51^ The obtained concentration (*C*_2_) was determined using formula:

1 $$C_{2}=\frac{C_{1}\times F_{H}}{F_{total}}$$

where *C*_1_ is the initial concentration of the test gas in the bottle, *F*_H_ is the gas flow rate, and *F*_total_ is the flow rate of the mixture of the test gas and ambient gas (synthetic air).

Using a mass flow controller, the flow rates of both gases (*F*_H_ and *F*_total_) were maintained at 200 standard cubic centimeters per minute (sccm) in the chamber to achieve optimum sensor response.

A heater placed under the sample was used to heat the sample from 20 to 250 °C, and the temperatures were controlled and monitored using a microcontroller. Samples were tested at relative humidity (RH) values of 10% and 50%, respectively. Here, the RH was measured using a hygrometer, and several levels of RH were generated using a bubble humidification setup as previously reported.^52^ Higher RH was generated using a bubbling system by passing air through DI water at room temperature and injecting it into a measurement chamber. RH was controlled continuously throughout the experiment using a specialized and calibrated SHT43 sensor (Digital Humidity Sensor with ISO17025 certification) placed next to the sample. A two-point probe was used to investigate the electrical properties of the device.

The gas response (*S*) was determined using the following formula:

2 $$S\left(\%\right)=\frac{R_{gas}-R_{air}}{R_{air}}\times 100\%$$

where *R*_gas_ is the resistance of the sample during gas exposure and *R*_air_ is the resistance of the sample in air. The error bar represents 10% of the gas response value.

Response and recovery times were determined from the dynamic response ranging from 10% to 90% and from 90% to 10% of the response value, respectively. The error for response and recovery times is ±0.5 s.^32^

## 3 Results and Discussion

### 3.1 Morphology and Compositional Analyses

Metal–organic-frameworks ZIF-71 dispersion was prepared in 2-propanol, as detailed in the experimental section. Figure 2a shows an image of the prepared dispersion. The dispersion was then drop-cast onto the CuO:Al film with Au-IDEs, as shown in Figure 2b. As the first step, the surface morphology of the CuO:Al film, as well as the ZIF-71-coated CuO:Al composite, was investigated by scanning electron microscopy (SEM). Figure 2c shows the corresponding SEM image of the CuO:Al film on a glass substrate. It reveals homogeneous intergranular structures that uniformly cover the entire glass slide substrate. The CuO:Al film exhibits densely packed, triangular-shaped nanograins, likely resulting from rapid thermal annealing at 650 °C for 60 s. These grains appear randomly oriented with no apparent preferential alignment, indicating isotropic growth of the grains during annealing.^14^ Additionally, SEM investigations on the morphology of ZIF-71-coated CuO:Al samples were conducted (Figure 2d, f, and g). Figure 2d displays a low-magnification SEM image of the CuO:Al surface after the initial 50 μL of ZIF-71 dispersion deposition. It shows coverage of the CuO:Al surface with metal–organic-framework ZIF-71 particles of rhombic dodecahedron morphology. Figure S1 displays a corresponding high-resolution SEM image, also showing these ZIF particles but still containing features of the underlying CuO:Al film. From this image, we can conclude that the particles are most likely ZIF-71 particles that adhere to the CuO:Al film. Furthermore, Figure S1 allows for a simple particle size estimation from four individual particles, containing a range of approximate particle sizes from 500 to 750 nm. Figure 2f and g shows high- and low-magnification SEM images of the sample surface after a complete cumulative 100 μL of ZIF-71 dispersion deposition. The comparison with the 50 μL of ZIF-71 sample from Figure 2d reveals an increase in CuO:Al surface coverage with ZIF-71 nanoparticles. Compositional analysis of CuO:Al was carried out using EDX characterization. Figure S2 shows the corresponding results. The bright-field SEM image from Figure S2 shows the analyzed region of approximately 5 μm × 4 μm in size. We calculated the composition of the film from Cu Lα1,2, O Kα1,2, and Al Kα1 signal images (Figure S2b–d). The following composition was found: O (43.6 at%), Al (0.1 at%), and Cu (56.3 at%).

**Figure 2:**
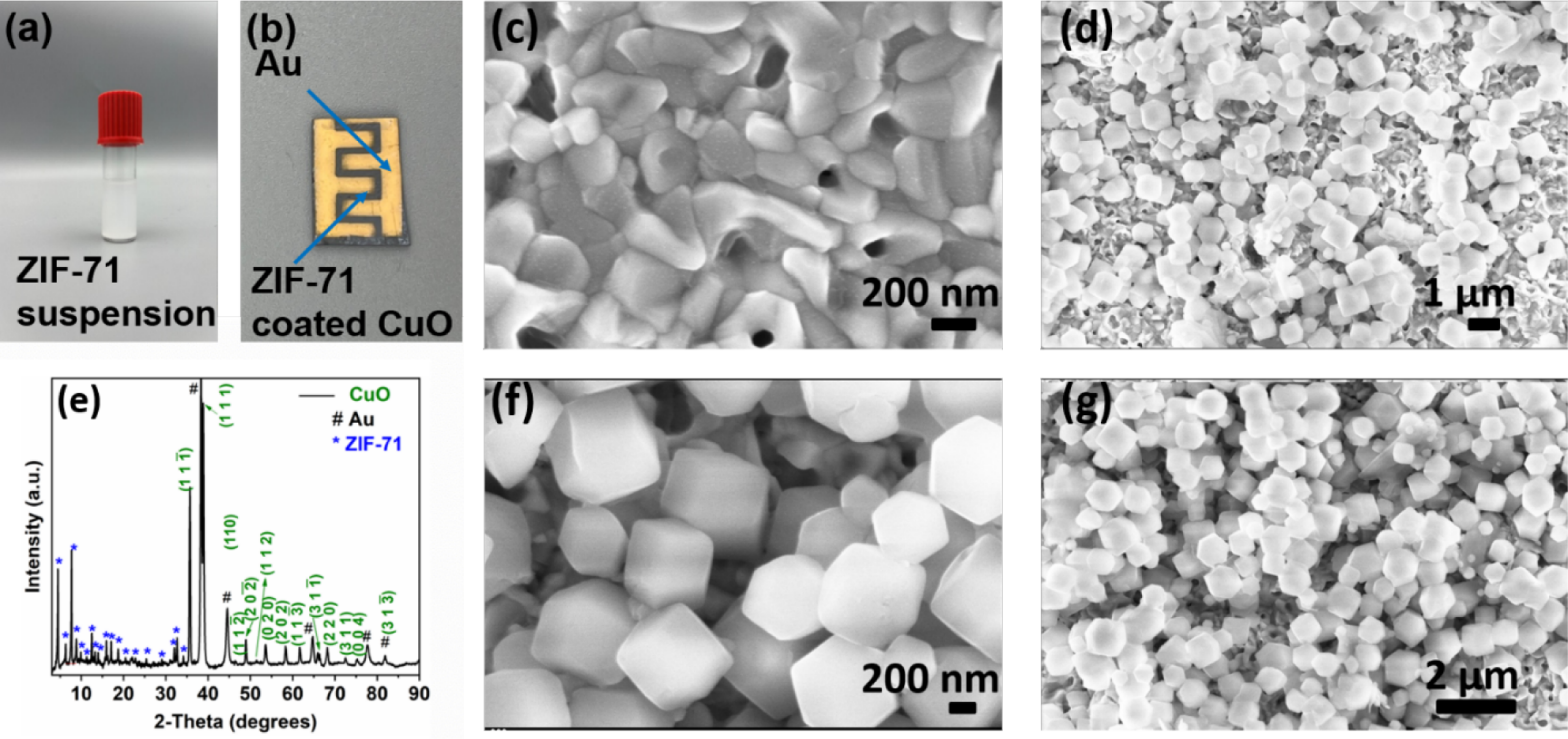
(a) A photographic image of ZIF-71 dispersion in 2-propanol. (b) Representative photographic image of the fabricated gas sensor device with metal–organic framework ZIF-71 dispersion drop-cast onto the CuO:Al metal oxide sensing layer and Au-IDEs. (c) Scanning electron microscopy (SEM) image of the CuO:Al film. (d) SEM image of the ZIF-71-coated CuO:Al sample after the initial 50 μL ZIF-71 dispersion deposition. (e) XRD pattern of the ZIF-71-coated CuO:Al film with Au contacts. (f,g) High- and low-magnification SEM images of the ZIF-71-coated CuO:Al film after a complete cumulative 100 μL ZIF-71 dispersion deposition.

All subsequent experiments were conducted on the CuO:Al films, which were almost fully covered with ZIF-71 nanoparticles deposited using a cumulative dispersion volume of 100 μL. Figure 2e shows the labeled XRD pattern of the ZIF-71-coated CuO:Al film. The primary reflexes of CuO were observed at 38.83° and 35.67°, corresponding to the (111) and (111̅) lattice planes of CuO (PDF card no. 1526990). This strongly indicates that the ZIF-71-coated CuO:Al film predominantly consists of the CuO phase. Well-defined Bragg reflections were detected across the entire measurement range and attributed to CuO (PDF card no. 1526990), Au (PDF card no. 1100138), and ZIF-71.^53,54^ The other five prominent reflexes correspond to Au, as shown in Figure 2e. The Au reflexes observed in the diffraction pattern are due to Au-IDEs on top of the structure for the electrical measurement. Figure S3 shows the XRD pattern of only Au-IDEs deposited on the CuO:Al film without the ZIF-71 deposition. It also displays distinct reflexes corresponding to CuO, which support the initial indication of a crystalline CuO phase being present in the CuO:Al film. No reflections of Al or related oxides (according to PDF card nos. 1000059, 1512488, and 1534642) were detected, which is most likely due to the low doping concentration of Al (0.1 at%) in oxide films. Additionally, in the XRD pattern of the ZIF-71-coated CuO:Al film shown in Figure 2e, multiple characteristic ZIF-71 reflexes were observed in the 2θ range from 4° to 35°. More distinct diffraction reflexes corresponding to ZIF-71 are visible in Figure S4, which shows the XRD pattern of the ZIF-71 nanoparticles alone. The primary characteristic diffraction reflexes of ZIF-71 appear at 2θ ∼4.42° and 7.67°, corresponding to the (110) and (211) planes, respectively.^53^ The crystallite size of the ZIF-71 nanoparticles was estimated using the Scherrer equation:^55^

3 $$d=\frac{K\times \lambda }{\beta \times \cos\theta }$$

where *d* is the crystallite size, *K* (∼0.940 for cubic-shaped particles) is the shape factor, λ (0.15406 nm) is the X-ray wavelength, β (in radians) is the full width at half-maximum (FWHM), and θ is the Bragg angle.

The most intense XRD peak corresponding to the (2 1 1) plane at 2θ ∼ 7.67° for ZIF-71 was selected for fitting to determine the FWHM. A Voigt function was employed for the peak fitting using the orthogonal distance regression pro iteration algorithm. An empirical approximation formula for the Voigt function was used to calculate the FWHM:^56^

4 $$\beta \approx 0.5346\times w_{L}+\sqrt{0.2166\times w_{L}^{2}+w_{G}^{2}}$$

where *w*_L_ is the Lorentzian width and *w*_G_ is the Gaussian width. The error for the β profile is ±0.02%.^56^

Using eq 4, the FWHM of the XRD peak corresponding to the plane (211) was estimated to be 0.002 radians. The crystallite size was then calculated by using eq 3, yielding an estimated value of approximately 57.28 nm. Crystallites are the smallest ordered regions within a material, separated by grain boundaries, which may even be partially amorphous. The Scherrer equation specifically measures the size of these coherent diffracting domains.^57^ A significant discrepancy was observed between the particle size (∼500 to 750 nm, estimated from SEM images) and the crystallite size (∼57.28 nm, estimated using the Scherrer equation). This suggests that ZIF-71 particles are composed of several smaller crystallites. These findings highlight the polycrystalline nature of ZIF-71 nanoparticles.^57^ The increased number of grain boundaries implies a greater number of active sites, which can enhance the material’s performance in sensing applications.

Figure 3a shows a Raman spectrum of the metal–organic framework ZIF-71-coated CuO:Al film. The presence of several characteristic peaks is indicative of the preserved structural integrity of the ZIF-71 framework. Peaks below 200 cm^–1^ are attributed to the ZIF-71 lattice framework, consistent with previously reported research.^58^ The peak at 443 cm^–1^ corresponds to the Zn–N stretching (υ) vibration. Peaks observed at 667, 1009, and 1063 cm^–1^ are assigned to the in-plane (δ) linker ring deformation of the imidazolate ligand, as reported in the literature.^59^ Additionally, peaks at approximately 295, 343, and 629 cm^–1^ are associated with the A_g_, B_1g_, and B_2g_ vibrational modes of the CuO film, respectively.^60^ Finally, the Raman bands at 1290 and 1240 cm^–1^ are attributed to the C–H vibrations, while the peak at 1331 cm^–1^ corresponds to the C=N stretching (υ) within the imidazolate ring, as verified by prior studies.^61^

**Figure 3:**
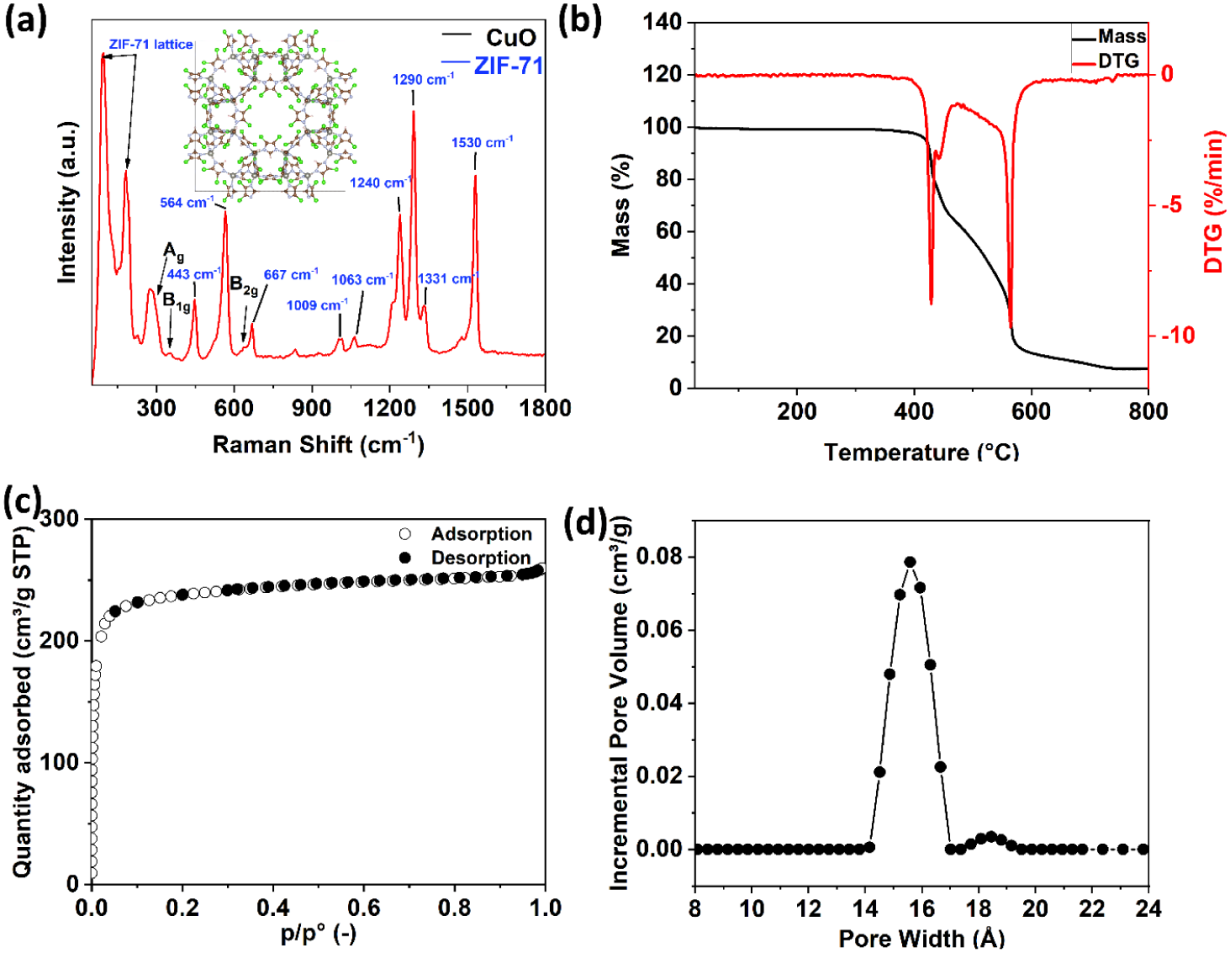
(a) Raman spectra for the metal–organic framework ZIF-71-coated CuO:Al film. (b) TGA and DTG profiles for the metal–organic framework ZIF-71. (c) N_2_ adsorption–desorption isotherm curves for ZIF-71. STP stands for standard temperature and pressure, *p* is the monitored pressure, and *p*_o_ is the saturated pressure. (d) Incremental pore volume curve as a function of pore width.

The thermal stability of ZIF-71 powder was evaluated using TGA. The weight loss (TG) and the derivative of the weight loss (DTG) are plotted in Figure 3b. The TGA shows negligible weight loss up to 400 °C, confirming that ZIF-71 is thermally stable up to 400 °C. A large weight loss around 415 °C is likely due to the decomposition of the ZIF-71 structure, which leads to the formation of ZnO. The total weight loss from 400 to 700 °C is 88%, which is higher than the theoretical mass loss of 76% when ZIF-71 converts into ZnO. This difference between the theoretical and experimental mass losses could be explained by the presence of 4,5-dichloroimidazole linkers coordinated to terminal zinc atoms at the crystal surface.^62^

A nitrogen (N_2_) adsorption–desorption isotherm was measured at 77 K to analyze the surface area and pore size distribution of the powder consisting of ZIF-71 particles. The N_2_ adsorption–desorption isotherm presented in Figure 3c exhibits a distinct, sharp inflection at low relative pressures (*p*/*p*_o_ < 0.1), characteristic of a Type-I isotherm under IUPAC classification, thereby confirming the presence of a microporous structure with pore diameters less than 2 nm.^63^ Figure 3d shows a Brunauer–Emmett–Teller (BET) pore size distribution, from which the surface area was calculated to be 969.9 ± 6.3 m^2^g^–1^ with an average pore size of 16.3 Å, which is in close agreement with the literature.^63^

XPS analyses were performed to study the surface chemistry, including chemical states and bonding environments of the elements present in a ZIF-71-coated CuO:Al film. Figure 4a displays the survey spectrum showing recognized signals from C and N present in imidazolate units, Zn cation, and Cl anion present in the organic ligand. No Cu or Al signals from the substrate are visible, indicating almost complete coverage of the substrate. A small O 1s signal is visible in the survey spectrum, indicating only very slight oxidation of the ZIF-71 surface even after prolonged storage of ZIF-71 in air. Note that prior to the XPS measurements, the samples were stored in a closed container in an air environment for several weeks. The high-resolution spectra are displayed in Figure 4b–g. Figure 4b shows the Zn 2p spectrum. It contains only the characteristic spin–orbit split peaks: Zn 2p_3/2_ at 1021.7 eV and Zn 2p_1/2_ at 1044.7 eV, with a typical splitting of 23.0 eV, confirmed the presence of a single Zn species. Figure 4c displays the C 1s line, which was fitted into two peaks at 286.8 eV (C 2) and 285.2 eV (C 1). The C 2 peak at 286.8 eV with 79% of the total C 1s peak intensity corresponds to the expected C–N/C=N and C–Cl bonds of intact ZIF-71,^64^ while the smaller C 1 peak at 285.2 eV with 21% of the total C 1s peak intensity indicates carbon not bonded to electron withdrawing partners, which would not be expected for intact ZIF-71. Figure 4d shows the fitted N 1s spectrum, revealing three components named N 1, N 2, and N 3. The component N 1 appearing at 397.6 eV is assigned to C–N bonding environments and exhibits 5% of the total nitrogen intensity. while the component N 2 at 399.7 eV is assigned to H–N bonding exhibiting 80% of the total nitrogen intensity.^64^ Furthermore, an additional nitrogen species (N 3) is observed at a higher binding energy of 401.5 eV, exhibiting 15% of the total N 1s peak intensity. The appearance of intensity at higher binding energy in the N 1s region (Zn–N ≈ 401.5 eV) was also observed in a study where the degradation of ZIF-71 upon exposure to X-rays at a synchrotron X-ray beamline was investigated.^65^ In this study, no effects of X-ray degradation were observed at the Zn 2p lines, supporting its use as a reference line for the binding energy. Moreover, Figure 4e shows the fitted Cl 2p spectra with four peaks named Cl 1, Cl 1′, Cl 2, and Cl 2′, corresponding to two spin–orbit-split doublets. The doublet Cl 2 and Cl 2′ is associated with the C–Cl 2p_3/2_ (201.3 eV)^66^ and C–Cl 2p_1/2_ (202.9 eV)^66^ bonds. and the doublet Cl 1 and Cl 1′ corresponds to Zn–Cl 2p_3/2_ (199.1 eV) and Zn–Cl 2p_1/2_ (200.7 eV) bonds,^65^ which appear and grow upon exposure to X-rays as already observed in the previously mentioned study.^65^ The expected Cl doublets for ZIF-71 make up 71% of the total intensity, while the Zn–Cl degradation product makes up 21% of the total Cl intensity. Thus, we also assign the C 1 species at 285.2 eV to the X-ray degradation products. The C 1 species corresponds to C–C and/or C–H species not bonded to nitrogen or chlorine. Initially, we did not expect and were not aware of such fast and severe degradation of ZIF-71 upon exposure to X-rays. For this reason, we remeasured the Cl 2p region after taking the complete first set of data for the ZIF-71 sample. The remeasured Cl 2p region is shown in Figure 4g. It clearly shows an increase of the doublet assigned to Zn–Cl bonds from 21% to 42% of the total Cl intensity and clearly shows that the X-ray degradation proceeds rather fast in relation to typical measurement times needed to take the XPS spectra. At this point, it is not clear whether the degradation is caused directly by the X-rays or by the creation of secondary electrons. The observation of a fast degradation, also in lab XPS experiments, points to the latter reason because the high brilliance of the X-rays at the synchrotron should cause much faster and more severe degradation of the sample if it were due to X-rays, while the difference in the creation of secondary electrons upon exposure to X-rays is more similar in a synchrotron and a lab experiment. Note that, as mentioned above in the Raman analysis, the presence of only the expected vibrations indicated an intact ZIF-71 layer, because here the ZIF-71 was not exposed to X-rays or electrons, indicating that the XPS analysis experiment started with an intact ZIF-71 layer. However, due to the fast degradation of the ZIF-71 under X-ray exposure, it appears to be not possible to obtain XPS spectra of intact ZIF-71 in a typical XPS lab experiment. This degradation of the ZIF-71 framework under high-dose X-ray exposure can be attributed to the presence of halogen (−Cl in this case), which facilitates the radical formation from C–Cl bonds. These radicals may initiate C–H or C–N activation and can also promote linker fragmentation.^67^ The O 1s line shows only a broad symmetrical peak at 532.5 eV, as shown in Figure 4f. This is due to slight oxidation of ZIF-71 under prolonged storage in air and could be indicative of oxidized carbon species like alcohols or ketones, oxidized nitrogen species, or possibly zinc hydroxides. The formation of ZnO (O 1s binding energy of about 530.7 eV) can be excluded.

**Figure 4:**
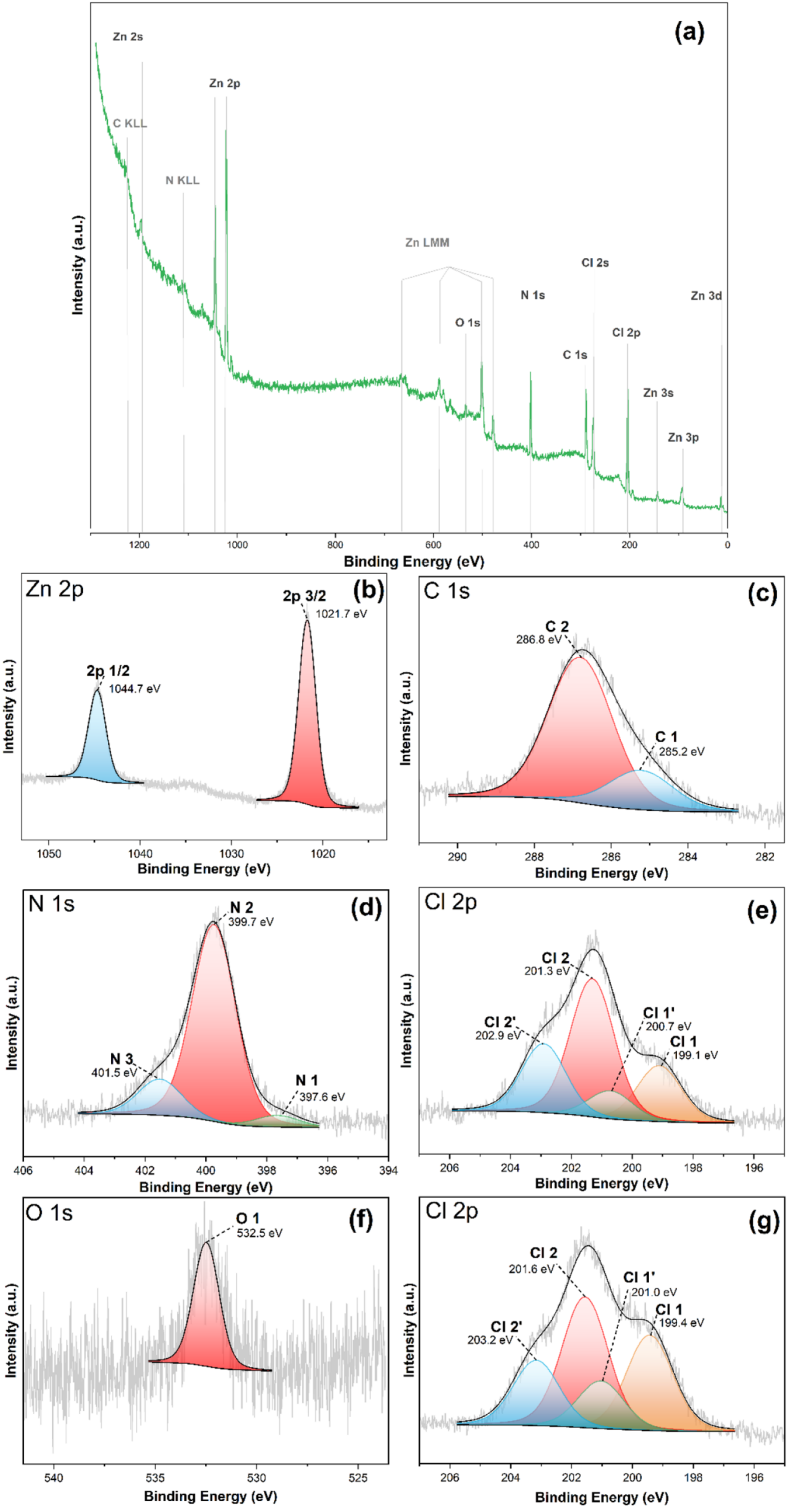
XPS spectra of the metal–organic framework ZIF-71-coated CuO:Al film: (a) Survey spectra showing XPS lines of all elements present. High-resolution (b) Zn 2p region. (c) C 1s region. (d) N 1s region. (e) Cl 2p region. (f) O 1s region. (g) Cl 2p region, second measurement.

The XPS elemental composition analysis gives 36.7 at% C, 23.8 at% Cl, 21.8 at% N, 15.7 at% Zn, and 2.1 at% O. The approximate 3:2 C-to-Cl or C-to-N ratio, as well as the approximate 1:1 N:Cl ratio, is consistent with the 4,5-dichloroimidazole units of ZIF-71, while the lower amount of Zn points to an excess of 4,5-dichloroimidazole units at the ZIF-71 surface, consistent with the observed higher weight loss in the TGA.

To study the location of the defect states, the activation energy (*E*_a_) of the CuO:Al film was assessed by analyzing current (*I*) measurements at different operating temperatures (*T*) under a 1 V bias voltage in an ambient environment. Figure S5 shows the corresponding ln­(*I*) against the 1/*T* Arrhenius plot. From the slope of the linear fit, an approximate activation energy (*E*_a_) was calculated. The slope approximated from the linear fit relates to *E*_a_ with the following equation:

5 $$Slope=-\frac{E_{a}}{k}$$

where *k* (eV/K) is Boltzmann’s constant and *T* (K) is the operating temperature.

The determined slope of the plotted curve is ∼−3.32 × 10^3^ K. Thus, the activation energy of the defect state is ∼0.28 eV. The corresponding activation energy relates to the defect level or hole trap states (*V*_Cu_).^68,69^ These trap states act as a copper shallow acceptor level compared to other metal oxides with deep acceptor levels, i.e., MgO.^69^

Band bending at the metal–semiconductor junction arises from the work function difference, leading to charge transfer across the interface.^70^ When Au contacts are formed with *p*-type CuO:Al, upward band bending occurs near the interface (Figure S6a,b). This configuration depletes electrons and accumulates holes, thereby lowering the barrier for hole injection and resulting in an Ohmic contact for the *p*-type semiconductor oxide. The extent of band bending depends on the difference between the metal’s work function and the semiconductor’s electron affinity.^71^ The bending modifies the work function and leads to the formation of a depletion layer with a width *W* at the junction. The resulting space charge region provides a low-resistance path for charge transport. Current–voltage measurements display linear behavior, confirming Ohmic contact, as shown in Figure S6c.

### 3.2 Sensing Results

CuO:Al (reference) and ZIF-71-coated CuO:Al samples were tested with a series of analyte gases at different operating temperatures to evaluate their sensing performance. Dynamic gas response measurements of the CuO:Al films were conducted. The dynamic response of the CuO:Al sensor (reference sample set) to 100 ppm of hydrogen at 250 °C shows a 45% response, as illustrated in Figure S7. The calculated response and recovery times were approximately 11 and 15 s, respectively. Figure 5a and b shows a comparative study of the gas response to the different analyte gases for CuO:Al-based gas sensors, without and with ZIF-71 coating on top, respectively. After applying the ZIF-71 layer to the CuO:Al film, the gas-sensing response to the tested analytes—hydrogen gas, *n*-butanol, 2-propanol, and acetone—improves at operating temperatures ≥200 °C, with a noticeable response observed at 200 °C compared to the uncoated CuO:Al sensor. This enhancement can be attributed to the synergistic interaction between the metal–organic framework ZIF-71 and CuO:Al at their interface, where selective adsorption by ZIF-71 at active sites leads to increased selectivity toward *n*-butanol and hydrogen gas at their respective optimal operating temperatures.^72,73^ In the case of *n*-butanol sensing, the most drastic increase in the gas response was observed. Here, the gas-sensing response (*S*) increases from approximately 0.1% to 11% when comparing the ZIF-71-coated sample with the bare CuO:Al reference sample at a temperature of 200 °C. This indicates an effective contribution of ZIF-71 to *n*-butanol sensing.

**Figure 5:**
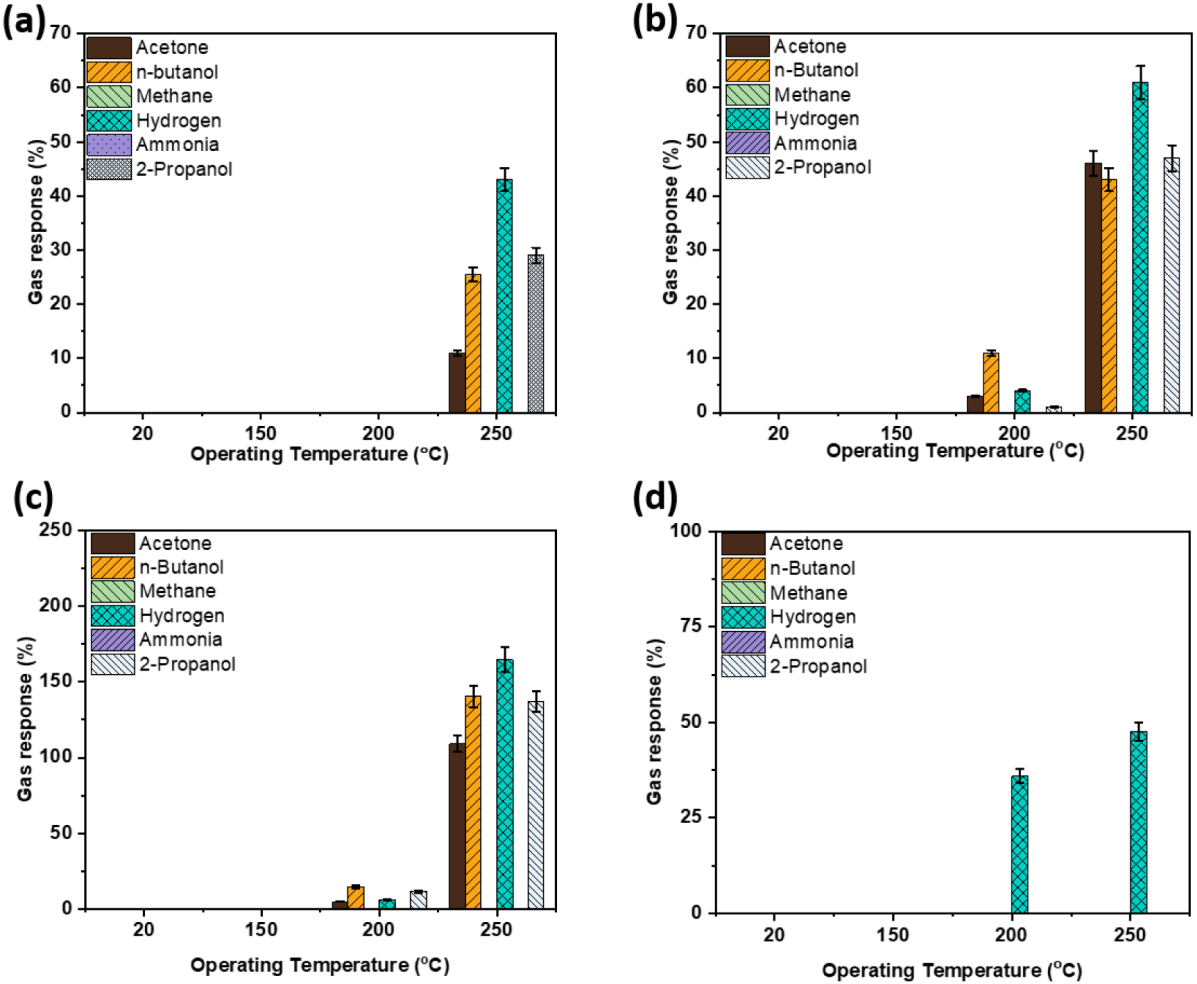
Gas response to a series of gases with a concentration of 100 ppm at different operating temperatures for the CuO:Al sample: (a) Without ZIF-71 coating, RH10%. (b) With metal–organic framework ZIF-71 coating, initial test, RH10%. (c) With metal–organic framework ZIF-71 coating after 21 days, RH10%. (d) With metal–organic framework ZIF-71 coating after 21 days, RH50%.

Figure 5b presents the sensor’s response to various gases at a concentration of 100 ppm. The maximum sensing response (*S* ∼ 60%) to hydrogen gas was observed at 250 °C. At low temperatures of 20 and 150 °C, no response was detected for any of the target gases. This lack of response is attributed to the adsorption of non-reactive molecular oxygen ionic species (O_2_^–^) on the sensor surface, which leads to slow reaction dynamics with the analytes at these temperatures.^74^

Between operating temperatures of 200–250 °C, the sensor responded to a 100 ppm concentration of VOCs and hydrogen gas. However, no response was observed for methane or ammonia at any tested temperature. This behavior may be due to the absence of oxygen functional groups in these analytes, resulting in only physisorption on the sensing surface.^15^ The sensor exhibited the highest sensing response toward *n*-butanol at 200 °C and toward hydrogen gas at 250 °C among the investigated temperatures. At 200 °C, the selectivity for *n*-butanol was approximately four times greater than that for hydrogen gas and five times greater than that for acetone.

To understand the sensor’s interaction with *n*-butanol, Gao et al.^75^ investigated the desorption rates of *n*-butanol in three different ZIFs: ZIF-8 (pore aperture of 3.4 Å), MAF-6 (pore aperture of 7.6 Å), and TIF-1 Zn (pore aperture of 11.7 Å). They found that the desorption rate decreases as the pore aperture decreases, with desorption temperatures of 230 °C for ZIF-8 and 130 °C for TIF-1 Zn. This clearly demonstrates the influence of pore size on desorption behavior.

Based on this trend, the desorption temperature of ZIF-71 (pore aperture 5.1 Å) is expected to fall between those of ZIF-8 and MAF-6, approximately in the range of 200–230 °C.^72^

Furthermore, Wang et al.^72^ reported that ZIFs with larger pore windows exhibit a higher affinity for *n*-butanol compared to acetone and smaller-chain alcohols. This supports the observation that ZIF-71 shows greater adsorption of *n*-butanol than acetone or 2-propanol prior to desorption.

To assess the gas-sensing response of the ZIF-71-coated CuO:Al sensor after subsequent measurement and storage time, a gas-sensing test 21 days after the initial measurement was conducted. Subsequently, this test was followed by another gas-sensing response test after an additional 21 days (in total, 42 days after the initial test). Figure 5c shows the ZIF-71-coated CuO:Al gas-sensing response (*S*) to the analyte gases at RH 10%, 21 days after the initial measurements. Overall, the gas-sensing response (*S*) increased in comparison to the initial measurements. At an operating temperature of 200 °C, the sensor exhibited a response comparable to the initial measurement, while at 250 °C, the response values for all test gases increased. The most significant increase in response, from approximately 43% to 141% at an operating temperature of 250 °C, was observed for *n*-butanol. This substantial enhancement is likely due to repeated measurements following the initial heating to 250 °C during the initial measurements. This process may have annealed the material, leading to an improved response.

Figure 5d shows the gas response of the ZIF-71-coated CuO:Al hybrid sensor to various test gases after 21 days of the initial measurements at higher relative humidity (RH = 50%). In the 200 to 250 °C operating temperature range, an increased selectivity toward 100 ppm hydrogen gas was observed. Figure 5c and d illustrate the effect of relative humidity on the response of the tested analytes (*n*-butanol, 2-propanol, acetone, hydrogen gas, ammonia, and methane gas). Increasing the relative humidity from 10% to 50% resulted in a pronounced deterioration of the sensor’s response to n-butanol across all operating temperatures. A greater deterioration in the gas-sensing response for all other analytes, except hydrogen gas, at higher RH is evident in Figure 5d. This effect may be attributed to the incomplete coverage of ZIF-71 on the surface of CuO:Al and the competitive adsorption of hydroxyl ions and oxygen molecules.

Additionally, the dynamic gas responses of the ZIF-71-coated CuO:Al film were measured 21 days after the initial measurements. In particular, dynamic responses to the tested analytes—acetone, 2-propanol, hydrogen gas, and *n*-butanol—were investigated. The response and recovery times determined from the corresponding dynamic measurement results are summarized in Table 1. Figure S8 shows the dynamic response to 100 ppm of acetone at 250 °C and 10% RH, 21 days after the initial gas-sensing test. It can be observed that the resulting ZIF-71 bridges, akin to the CuO:Al film, exhibited a favorable resistance of approximately 4 kΩ (in air) at a low bias voltage of 6 mV. The test gas was applied multiple times for ∼30 s with similar response values of ∼109% observed for 100 ppm acetone. This demonstrates notable repeatability of the acetone response at 250 °C over three consecutive cycles. This result highlights the consistency and reliability of the fabricated gas sensor tested at a 21-day interval following the initial measurement. Figure S9 shows the ZIF-71-coated CuO:Al film’s dynamic response to 100 ppm 2-propanol gas at 250 °C. Here, a maximum gas response of ∼138% was observed. The measured response and recovery times are 18 and 73 s, respectively. Additionally, Figure 6 displays the ZIF-71-coated CuO:Al film’s dynamic response to 100 ppm hydrogen gas at 250 °C at different RH levels. Here, a maximum gas response of ∼164% at RH 10% and ∼47% at RH 50% was observed. In addition to the dynamic responses to hydrogen gas and 2-propanol, the dynamic response to *n*-butanol was investigated. Figure 7 shows the dynamic response to 100 ppm of n-butanol at 250 °C with a maximum gas response of ∼141%. The corresponding response and recovery times are 19 and 105 s, respectively. One reason for the relatively slow response and recovery times in both cases (2-propanol and *n*-butanol) is the relatively slow diffusion of the test gas through the ZIF layer to the sensing material.^10^

**Table 1:** Response and Recovery Times Calculated 21 Days after the Initial Measurements at 250 °C at RH = 10%

Test gas	Operating temperature, °C	Response time, s	Recovery time, s
Acetone	250	14	52
2-Propanol	250	18	73
Hydrogen	250	2	11
*n-*Butanol	250	19	105

**Figure 6:**
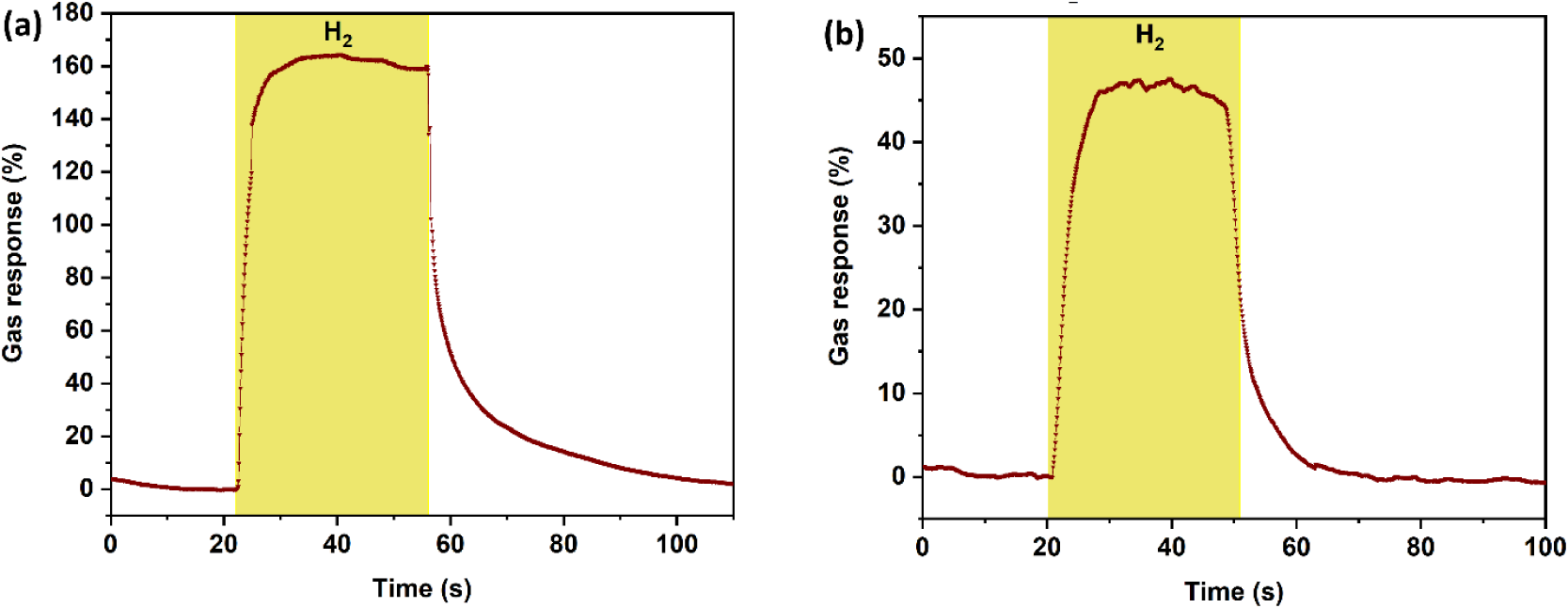
Dynamic response of the metal–organic framework ZIF-71-coated CuO:Al film-based hybrid sensor to 100 ppm hydrogen gas at 250 °C, measured 21 days after the initial measurements at different relative humidities: (a) RH = 10%. (b) RH = 50%.

**Figure 7:**
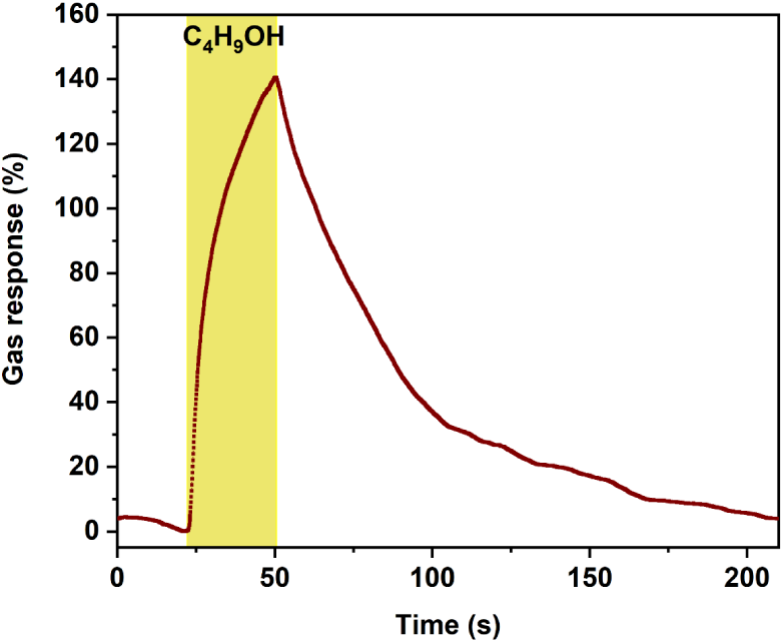
Dynamic response of the metal–organic framework ZIF-71-coated CuO:Al film-based hybrid sensor to 100 ppm *n*-butanol at 250 °C, measured 21 days after the initial measurements.

As summarized in Table 1, the developed hybrid sensors exhibited high response and recovery times to VOCs, while demonstrating fast adsorption and desorption dynamics for 100 ppm hydrogen gas.

Using the Boltzmann distribution and the Poisson equation, the dependence of resistance on surface band bending in the *p*-type CuO:Al sensing layers is analytically derived, highlighting surface-dominated transport^76^ of adsorbed species as shown in eq 6.

6 $$R_{air}\approx e^{-qV^{air}/2kT}$$

The gas-sensing response can be defined as the ratio of the resistance of the CuO:Al surface in the presence of gas (*R*_gas_) to the resistance of the CuO:Al surface in the absence of test gas (*R*_air_). The relationship between the sensor signal and the resistance change in terms of band bending as shown:

7 $$\mathrm{Gas}‐\mathrm{sensing response},S=\frac{R_{gas}}{R_{air}}\approx \frac{e^{qV^{air}/2kT}}{e^{qV^{gas}/2kT}}\approx e^{-q\Delta V/2kT}$$

This can be related to the band bending (*q*Δ*V*)^76^ as shown in eq 8. The effect of band bending and the related change in affinity can be understood with the help of eqs 8 and 9:

8 $$q\Delta V=-2kT\times \ln\left(\frac{R_{gas}}{R_{air}}\right)$$

The change in electron affinity can be extracted from the band bending equation as follows:

9 Δχ=ΔΦ−qΔV

where *T* is the operating temperature, Δχ is the change in electron affinity, and ΔΦ is the change in the work function of the CuO:Al sample.

The effect of relative humidity can be understood using eq 10. The exposure of the CuO:Al sample set to the target gas (e.g., *n*-butanol) in the presence of relative humidity has a complex effect. It is assumed that the band bending decreases (surface resistance increases) due to the decrease of ionosorbed oxygen replaced by the hydroxyl group.^76^ The exposure of water vapor to the oxygen adsorbed on the CuO surface leads to the formation of dipoles as follows:

10 $$O_{\left(ads\right)}^{-}+H_{2}O_{\left(vap\right)}+2Cu_{\left(site\right)}+h^{+}⇋2\left(Cu_{\left(site\right)}^{+}-OH^{-}\right)+S$$

where O^–^_(ads)_ is an ionosorbed oxygen species, H_2_O_(vap)_ is the water vapor in the atmosphere, Cu_site_ is an atomic Cu site on the surface, h^+^ is the consumed hole, (Cu^+^_(site)_ *–* OH^*–*^) is the formed terminal hydroxyl group (electric dipole), and *S* is a surface site for oxygen chemisorption.

The electric dipole contribution to the work function improves electron affinity by making it more difficult for electrons to escape from the sample surface.^76^ Thus, by increasing the relative humidity, the dominant electron-trapping species shifts from adsorbed oxygen to water vapor,^77,78^ leading to an increase in resistance and, consequently, a decrease in sensing response. Figure 5(d) illustrates a remarkable selectivity toward hydrogen gas at a higher RH of 50% after 21 days from the initial measurements.

After a total of 42 days following the initial measurement of the ZIF-71-coated CuO:Al sensor, another gas-sensing response test was performed at RH 10%, and the corresponding results are presented in Figure S10. At an operating temperature of 200 °C, the sensor exhibited a response comparable to that of the previous measurements. At 250 °C, the response values for VOCs are similar to the results obtained after 21 days, while a slight decrease in the response value for hydrogen gas was observed. In summary, the subsequent measurements repeated 21 and 42 days after the initial measurements showed similar response trends, suggesting that the sensor performance remained relatively stable over this period. These observations imply that thermal exposure during the initial testing could have contributed to the stabilization of the sensing characteristics.^79^

Figure 8 presents the comparative gas-sensing responses for all test gases and VOCs measured at 21 and 42 days after the initial test, which was conducted at 250 °C for 100 ppm hydrogen gas and VOCs under RH 10%. The subsequent results, measured 21 or 42 days after the initial measurements had stabilized, are attributed to the preheating effect.^79^ An approximately 2–3-fold increase in gas-sensing response was observed after 21 days, which may be attributed to the effects of thermal treatment by aging the sensor at higher temperatures.

**Figure 8:**
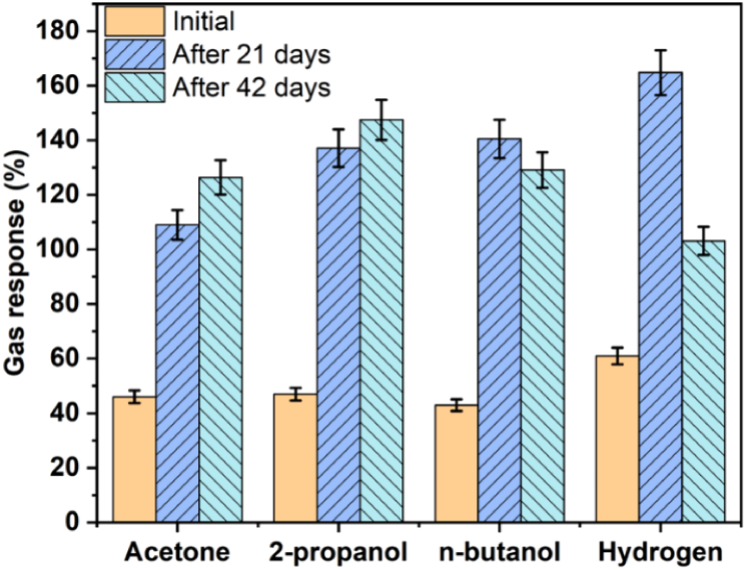
Gas response of the metal–organic framework ZIF-71-coated CuO:Al film-based hybrid sensor to a series of gases with a concentration of 100 ppm at RH 10% at 250 °C.

Table 2 provides a detailed comparison of the presented gas-sensing results with similar studies employing ZIF-layer coatings on semiconducting oxide films with varying morphologies. Notably, even after ZIF coating, a sensor from this study exhibited high efficiency, characterized by rapid response and recovery times (2 s/11 s) for hydrogen gas and a significant sensitivity enhancement (∼110 times) for *n*-butanol. Furthermore, the sensor demonstrated repeatable, temporally stable, and consistently selective performance for hydrogen gas, even under high relative humidity conditions (RH 50%).

**Table 2:** Comparative Study of Presented Sensing Results with Other Reported Literature Results Using a Similar Material Combination

Material (type) ref.	Concentration, test gas	Response value, *S*(%)	Enhancement in sensing response after ZIF-71 coating (*S*_ZIF‑71coated_/*S*_uncoated_)	Response/recovery time (s)	Relative humidity (%)	Operating temperature (°C)
ZnO (nanorod arrays)^45^	50 ppm, hydrogen	∼100	0.8	-	-	250
ZnO@ZIF-71 (nanorod arrays)^45^		∼80		-	-	
ZnO (nanorod arrays)^36^	10 ppm, ethanol	2.59	∼4	419.4/988.2	-	150
ZnO@ZIF-71 (nanorod arrays)^36^		13.4		194.3/442.1		
ZnO (nanorod arrays)^36^	5 ppm, acetone	20.6	∼1.8	373.3/772.4	-	150
ZnO@ZIF-71 (nanorod arrays)^36^		38.9		195.9/535.5	-	
WO_3_ (nanorod arrays)^35^	20 ppm, ethanol	27	∼6	-	-	250
WO_3_@ZIF-71 (flower-like)^35^		163				
WO_3_ (nanorod arrays)^35^	20 ppm, acetone	47	∼2.1	-	-	250
WO_3_@ZIF-71 (flower-like)^35^		101				
CuO/1%PMA (nanofibers)^80^	100 ppm, ethanol	66	-	5/4	30	240
CuO (nanowires)^16^	2500 ppm, hydrogen	100	-	25/14	0	300
		115		73/80	50	
CuO:Al (intergranular grains) (this work)	100 ppm, hydrogen	42	∼1.4	11/14	10	250
ZIF-71-coated CuO:Al (rhombic dodecahedron) (this work)		61		2/11	10	
CuO:Al (intergranular grains) (this work)	100 ppm, *n*-butanol	∼0.1	∼110	-	10	200
ZIF-71-coated CuO:Al (rhombic dodecahedron) (this work)		11		∼23/110	10	

### 3.3 Selectivity of ZIF-71-Coated CuO:Al

The transport mechanism of gas molecules through the MOF-ZIF-71 layer at the CuO:Al interface can be well understood by knowing the flow regime of gas molecules. The mean free path (λ) of the gas molecules can be defined:^81^

11 $$\lambda =\frac{kT}{\surd 2\pi r_{ij}^{2}p}$$

where *T* is the operating temperature, *r*_*ij*_ is the collision diameter, and *p* is the atmospheric pressure.

The concentration of test gases (100 ppm) is negligible compared to the background air. Consequently, the number density of the background air dominates, rendering the mean free path independent of the small mole fraction of the test gases.

Using eq 11, the mean free paths of *n*-butanol and hydrogen gas were calculated at 200 and 250 °C. The results are 62.5 and 136 nm at 200 °C and 69 and 150 nm at 250 °C, respectively.

The gas transport regime of the target gases can be classified into three classes (Knudsen diffusion regime, translational diffusion regime, and molecular diffusion regime) based on the Knudsen number (*K*_n_), which can be calculated using the relation:^81,95^

12 $$K_{n}=\frac{\lambda }{r_{p}}$$

where *r*_p_ is the pore aperture size of ZIF-71.

The Knudsen numbers (*K*_n_) for *n*-butanol and hydrogen gas at 200 and 250 °C were calculated using eq 12. The results are approximately 122 and 135 for *n*-butanol and 267 and 294 for hydrogen gas, respectively. These values indicate that the transport mechanism for both gases at both temperatures falls within the Knudsen diffusion regime (*K*_n_ > 10).

SEM images of the ZIF-71-coated CuO:Al film revealed that the ZIF-71 nanoparticles nearly fully covered the CuO:Al surface as a continuous film. However, the ZIF-71 layer is not fully free of pinhole defects. Transport through grain boundaries and other small defects may locally reduce the value of *K*_n_ in certain regions. It can be concluded that the intrinsic transport of *n*-butanol and hydrogen through the ZIF-71 pores at both temperatures would dominate and proceed via Knudsen diffusion. Nevertheless, the contribution from translational or molecular diffusion may arise locally at grain boundaries and defects.

The large pore aperture size of ZIF-71 (∼5.1 Å),^72^ which is close to the kinetic diameter of VOCs such as 2-propanol (∼4.7 Å),^82^ *n*-butanol (∼5.0 Å),^75^ and acetone (∼4.6 Å),^83^ allows competitive diffusion of these molecules through its pores. The comparable size of *n*-butanol, acetone, and 2-propanol to the pore aperture size provides easy access to them due to the molecular sieving effect. However, *n*-butanol has a stronger affinity for ZIF-71 adsorption sites compared to smaller molecules such as acetone and 2-propanol due to the longer chain of carbon atoms providing interaction sites for van der Waals interactions within the ZIF-71 framework.^84^ Wang et al.^72^ calculated adsorption energies for acetone and *n*-butanol on ZIF-71, showing a higher adsorption energy for *n*-butanol over acetone on the ZIF-71 framework. The effect of polarizability also influences the interaction strength between the adsorbate and the adsorbent. Due to the high polarizability of *n*-butanol (∼8.57)^85^ over acetone (∼6.27)^85^ and 2-propanol (∼6.67),^85^ *n*-butanol exhibits stronger van der Waals interactions with the ZIF-71 framework. This leads to its higher adsorption on ZIF-71, facilitated by effective surface interactions.^75^ Consequently, *n*-butanol reaches the interface more efficiently, resulting in higher sensitivity compared to acetone and 2-propanol. The sensor showed good sensing performance at lower temperatures (∼200 °C), attributed to the strong van der Waals interaction and hydrogen bonding between the hydroxyl group in *n*-butanol and the functional groups (−Cl) present in the MOF, resulting in high adsorption of *n*-butanol and leading to an enhanced sensing response. The low kinetic energy of *n*-butanol at 200 °C facilitates strong interaction with the ZIF-71 framework, allowing it to readily reach the heterojunction interface. In contrast, hydrogen, due to its smaller size, diffuses rapidly at higher temperatures, resulting in an increased sensing response. Figure 9 presents a schematic illustration that elucidates the properties and structural features of ZIF-71 relevant to gas-sensing applications. These properties collectively contribute to the selective adsorption and diffusion of specific analytes, thereby enhancing gas selectivity and sensor performance. The crystal structure of ZIF-71 illustrating its sodalite topology composed of Zn ions tetrahedrally coordinated with 4,5-dichloroimidazolate linkers.

**Figure 9:**
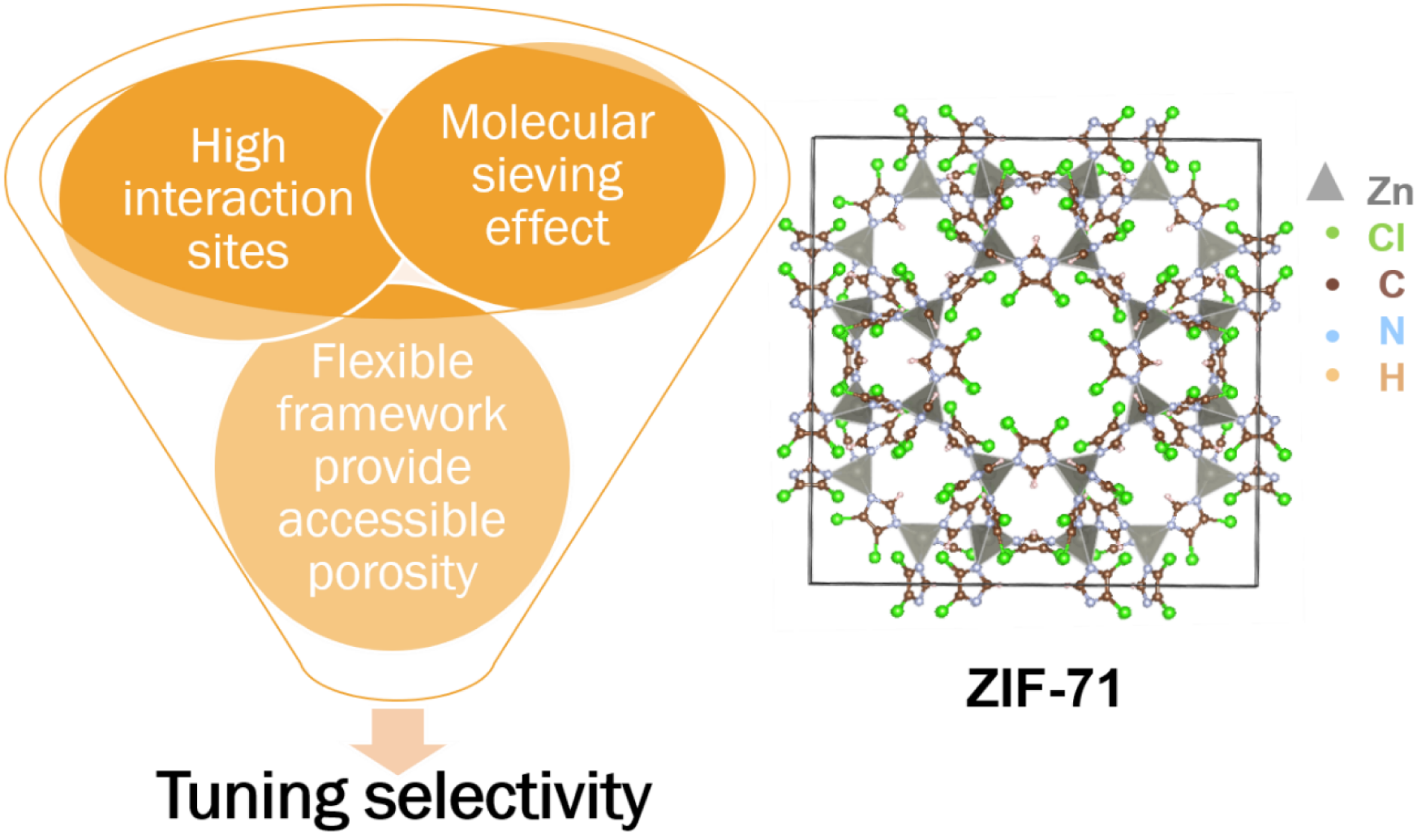
Schematic for tuning the selectivity using porous crystalline materials. Crystal structure of the metal–organic framework ZIF-71 visualized using VESTA.^94^ Adapted with permission from ref.^53^ Copyright 2008 Science.

### 3.4 Proposed Sensing Mechanism

ZIF-71-coated CuO:Al has a significant effect on the gas-sensing performance toward VOCs such as acetone, *n*-butanol, and 2-propanol, as well as gases such as hydrogen, methane, and ammonia. To understand the mechanism of enhanced gas selectivity for *n*-butanol at low temperature (200 °C) and hydrogen gas at higher temperature (250 °C), the model used for the interaction of analytes and the sensing surface is presented in Figure S11. For gas sensing on CuO, the model consists of three processes: sorption, diffusion, and desorption. The study by Wei et al. indicated that variation in adsorption and desorption of tested analytes is a key factor in the gas-sensing response.^86^ As discussed in the previous section, *n*-butanol exhibits a stronger affinity toward the ZIF-71 framework compared to acetone and 2-propanol, due to its higher polarizability, greater adsorption energy, and longer carbon chain. The integration of ZIF-71 with CuO:Al enhances the interaction of *n*-butanol at the ZIF-71/CuO:Al interface, owing to its strong molecular affinity. The reported study of analyte affinity for effective adsorption on ZIFs shows better *n*-butanol affinity toward ZIF-71 compared to acetone and other short-chain alcohols.^72^ The cavity size of ZIF-71, which has been reported to be slightly larger than that of the acetone molecule, leads to a weak affinity of acetone for the weakly polar composition of ZIF-71. This is due to the high dipole moment of acetone compared to n-butanol.^73,87^ Thus, *n*-butanol is expected to have the highest affinity toward ZIF-71 compared to acetone and short-chain alcohols. The synergistic effect of the CuO:Al contribution to *n*-butanol sensing and ZIF-71’s strong affinity toward *n*-butanol adsorption leads to selective *n*-butanol sensing at 200 °C, just below its desorption temperature.

Metal oxide semiconductors, such as ZnO and TiO_2_, in their pure composition (i.e., without doping), often exhibit *n*-type behavior due to the presence of inherent oxygen vacancies.^88,89^ However, there are a few metal oxides, such as CuO, that exhibit *p*-type semiconductor behavior due to inherent defects (metal interstitial defects), where Cu interstitial defects lead to the formation of acceptor states above the valence band. In general, in gas sensing by metal oxide semiconductors, adsorption of oxygen molecules from the atmosphere activates the inherent defect states and is considered to be the origin of gas sensing.^90^ The gas-sensing mechanism for reducing gases, such as *n*-butanol, 2-propanol, and hydrogen, can be explained by the ionosorption–desorption model. When the sensor is exposed to atmospheric air, oxygen molecules are adsorbed on the sensing surface and ionized to different oxygen species, depending on the operating temperature,^91^ as shown in eqs 13–18:

13 $$O_{2\left(gas\right)}↔O_{2\left(ads.\right)}$$

14 $$O_{2\left(ads.\right)}↔O_{2\left(ads.\right)}^{-}+h^{+}$$

15 $$O_{2\left(ads.\right)}^{-}↔2O_{\left(ads.\right)}^{-}+h^{+}$$

16 $$O_{\left(ads.\right)}^{-}↔O_{\left(ads.\right)}^{2-}+h^{+}$$

17 $${\left(C_{4}H_{9}OH\right)}_{ads.}+12O_{ads.}^{-}+12h^{+}\to 4CO_{2}+5H_{2}O$$

18 $$e^{-}+h^{+}↔\mathrm{null}$$

The above equations show the origin of the oxygen ionic species at different temperatures. eq 14 shows the origin of molecular oxygen ionic species at operating temperatures below 100 °C. eq 15 shows the origin of monoionic oxygen species in the temperature range 100 °C < *T* ≤ 300 °C, and eq 16 shows the origin of bionic oxygen species at operating temperatures above 300 °C. Figure S11 shows that a high potential barrier and a hole accumulation layer are formed due to the adsorption of oxygen ions on the sensing surface by surface traps, which leads to upward band bending and results in a decrease of the resistance of the accumulation layer. After *n*-butanol exposure to the sensing surface at 200 °C, the adsorbed oxygen species react with *n*-butanol, and the released electrons recombine with the holes in the bulk, as shown in eqs 17 and 18. This leads to a decrease in the hole concentration in the accumulation layer, causing the band to bend downward (*q*Δ*V* = Δφ) and increasing the resistance of the accumulation layer.^11^ As described in previous studies,^92^ the contribution to the sensor resistance consists of the contact resistance between the electrodes, the sensing surface contribution, the bulk contribution, and the hole accumulation layer resistance contribution. The contribution of each resistance depends on the sensor morphology and other factors.^93^

## 4 Conclusions

In summary, CuO:Al thin films were synthesized on glass substrates by using an SCS approach. A dispersion of ZIF-71 was drop-cast onto the synthesized CuO:Al thin films to create an organic/inorganic hybrid structure. XRD analysis confirmed the crystalline structure and good distribution of ZIF-71 nanoparticles. SEM images provided insights into the surface morphology, revealing intergranular structures of the CuO:Al film and triangular crystallites due to the thermal annealing effect. XPS reveals chemical composition of the ZIF-71 with the elemental composition values, 36.7 at% C, 23.8 at% Cl, 21.8 at% N, 15.7 at% Zn, and 2.1 at% O, indicating a C-to-Cl ratio of 3:2 and 1:1 N-to-Cl ratio, consistent with linker and lower presence of Zn points in ZIF-71. It also validate the higher weight loss observed in the TGA studies. XPS validated the successful coating of ZIF-71 on the CuO:Al film. Raman spectroscopy further validated the structural characteristics, identifying molecular vibrations corresponding to the CuO and ZIF-71 phases. ZIF-71-coated CuO:Al grains as organic/inorganic hybrid structure exhibit good sensing performance for two analytes, *n*-butanol and hydrogen gas, at two different operating temperatures, 200 and 250 °C, respectively. The fabricated hybrid sensors exhibited good temporal and chemical stability under ambient conditions and in the presence of analytes with good repeatability. The dispersion of ZIF-71 nanoparticles on the CuO:Al film provides suitable active sites for *n*-butanol over other smaller carbon chain alcohols and acetone. This can be explained by the high affinity of *n*-butanol for the ZIF-71 adsorption sites just before the desorption temperature. The selectivity for *n*-butanol at 200 °C was approximately four times higher than that for hydrogen gas and about five times higher than that for acetone during the initial measurements. The comparison between the gas-sensing response of the ZIF-71-coated CuO:Al and the reference sample (without ZIF-71 coating) shows an enhancement in the gas-sensing response to the target analytes at their respective temperatures. Fast response and recovery times were observed for hydrogen gas at 2 and 11 s, respectively. The proposed gas-sensing mechanism elucidates the interaction between analytes and ZIF-71, incorporating the diffusion of analyte molecules onto the CuO:Al surface. These results highlight the critical role of MOF ZIF-71 active sites in facilitating target analyte interactions, thereby enhancing gas-sensing performance of the humidity-compatible chemiresistive hybrid sensor. Future studies are necessary in order to investigate the influence of various ZIF-71 concentrations under different conditions. Functionalized ZIF-coated metal oxide-based hybrid sensors exhibit synergistic effects on the physicochemical properties of the composite material, enabling diverse applications such as fuel leak detection systems, battery safety early warning systems, and monitoring VOCs in industrial environments.

## Supplementary Material





## Notes

Particle size determination of ZIF-71 nanoparticles using SEM; the SEM image of the investigated area; and the composition images of the CuO:Al film using EDX mapping. X-ray diffraction pattern of the CuO:Al film with electrical Au contacts; X-ray diffraction pattern of ZIF-71 nanoparticles. the Arrhenius plot of ln­(*I*) vs 1/*T* for the calculation of activation energy; the energy band diagrams of Au and CuO; current–voltage measurement of Au-IDE contacts to *p*-type CuO:Al sample; dynamic response of the CuO:Al -based sensor to 100 ppm of hydrogen gas at 250 °C; dynamic response of the ZIF-71-coated CuO:Al film to 100 ppm acetone at 250 °C at RH = 10%, measured 21 days after the initial measurements; dynamic response of the ZIF-71-coated CuO:Al film-based hybrid sensor to 100 ppm 2-propanol at 250 °C, measured 21 days after the initial measurements; gas-sensing response to a series of gases with 100 ppm concentration at different operating temperatures for ZIF-71-coated CuO:Al films: after 42 days from the initial measurement at RH 10%; gas-sensing mechanism of the CuO:Al illustrated energy band diagrams toward *n*-butanol and other reducing gases; the absorption of oxygen species from ambient air; and in the presence of target gas (*n*-butanol) (PDF)
Data ZIF-71 (CIF)
