# Supplementary material for: ZIF-71-Coated CuO:Al with Enhanced Gas-Sensing Performance for n‑Butanol and Hydrogen
Source: ACS Appl Electron Mater. 2025 Nov 12;7(22):10198–215. doi: 10.1021/acsaelm.5c01659 (PMC12661656; doi:10.1021/acsaelm.5c01659)
Supplement: Supplementary file 1 [file el5c01659_si_001.pdf]

Supporting information

ZIF-71-coated CuO:Al with enhanced gas-sensing performance for n-butanol and hydrogen

Rajat Nagpal [1,2], Masaya Sugihara [3], Cristian Lupan [2], Tim Tjardts [4], Nahomy Meling-Lizarde [4], Thomas Strunskus [4,5], Haoyi Qiu [1], Rainer Adelung [1,5], Rob Ameloot [3], Oleg Lupan [1,2*]*

[1] Chair for Functional Nanomaterials, Department of Materials Science, Faculty of Engineering, Kiel University, Kaiserstraße 2, D-24143 Kiel, Germany

[2] Center for Nanotechnology and Nanosensors, Department of Microelectronics and Biomedical Engineering, Faculty CIM, Technical University of Moldova, 168 Stefan cel Mare str., MD-2004, Chisinau, Republic of Moldova

[3] Centre for membrane separations, Adsorption, Catalysis, and Spectroscopy, KU Leuven, 3001 Leuven, Belgium

[4] Chair for Composite Materials, Department of Materials Science, Faculty of Engineering, Kiel University, Kaiserstraße 2, D-24143 Kiel, Germany

[5] Kiel Nano, Surface and Interface Science KiNSIS, Kiel University, Christian Albrechts-Platz 4, 24118, Kiel, Germany

*Corresponding authors:
Rajat Nagpal (rajat@doctorat.utm.md, rajr@tf.uni-kiel.de),
Oleg Lupan (ollu@tf.uni-kiel.de, oleg.lupan@mib.utm.md)

KEYWORDS: ZIF-71; metal-organic-frameworks, n-butanol; polarizability; adsorption; affinity; synergistic; dipole; sensor

[illegible]

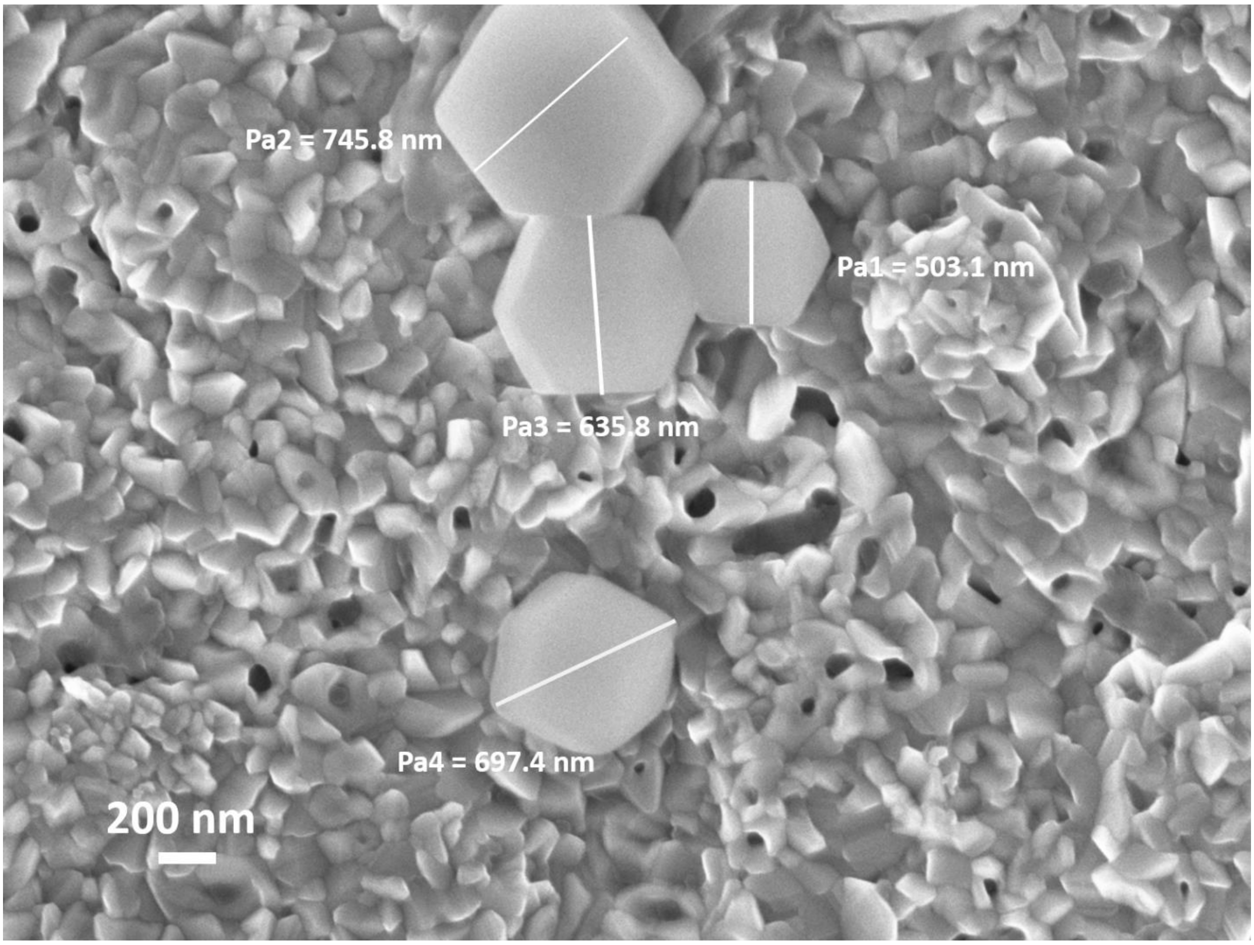

Figure S1. Particle size determination of MOF ZIF-71 nanoparticles using SEM, showing an average diameter in the range 500 to 750 nm.

Compositional analysis using EDX

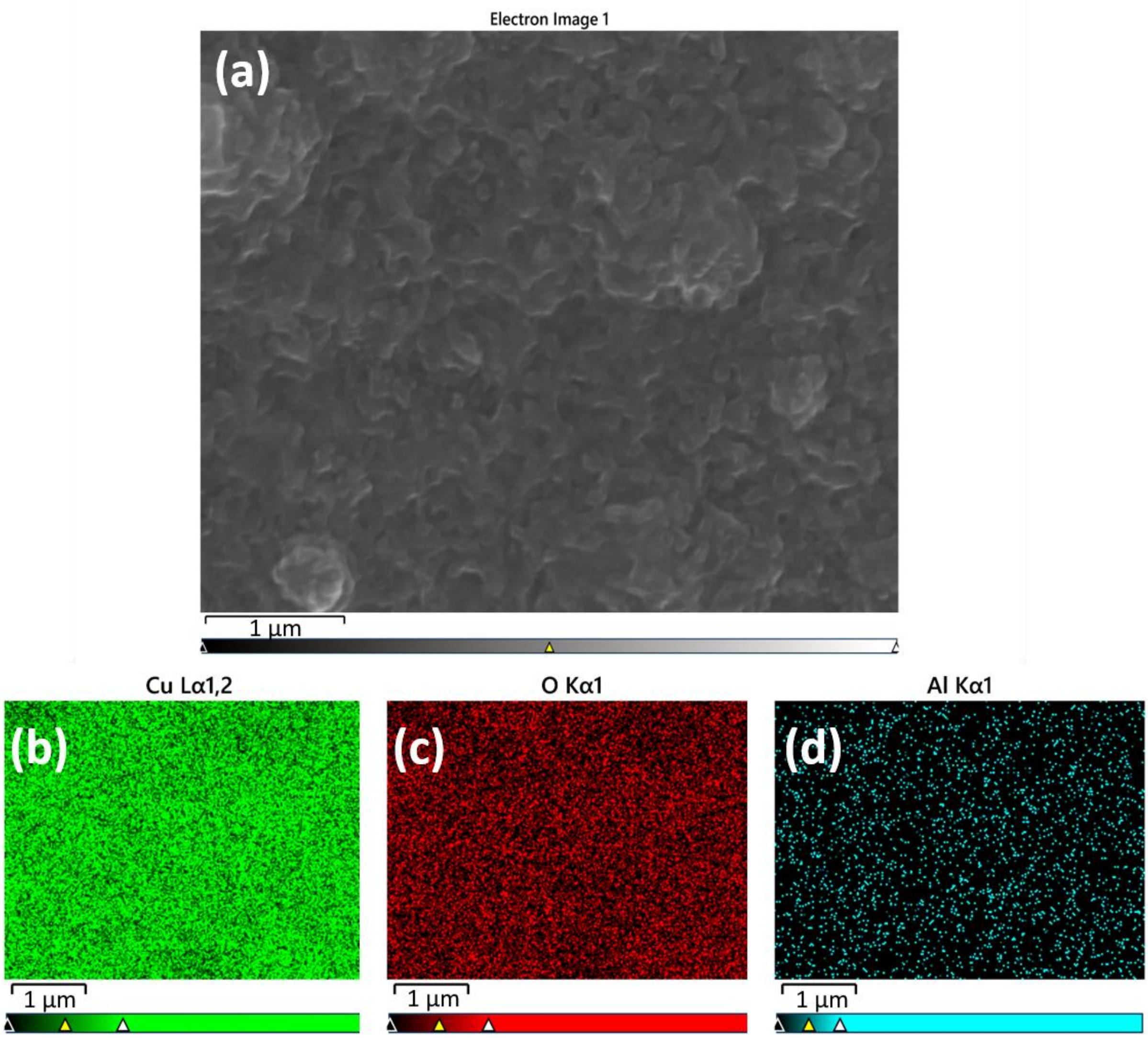

Figure S2. (a) The SEM image of the investigated area; and the composition images of the CuO:Al film using EDX mapping: (b) Cu distribution. (c) O distribution. (d) Al distribution.

Structural properties using XRD

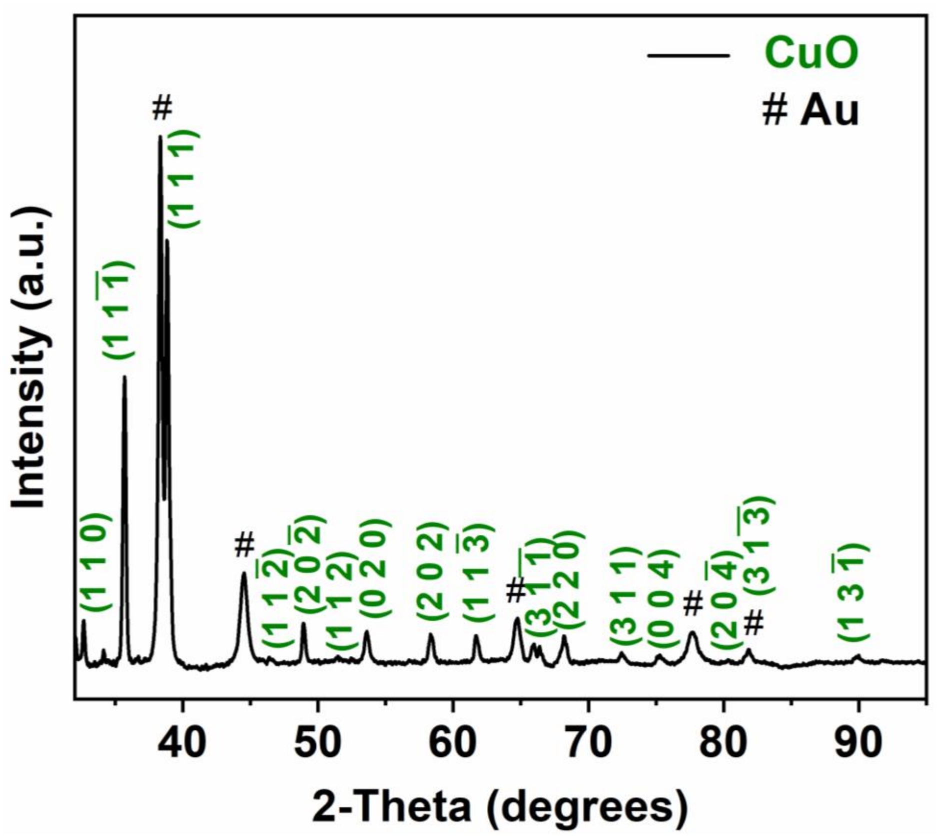

Figure S3. X-ray diffraction pattern of the CuO:Al film with electrical Au contacts on top.

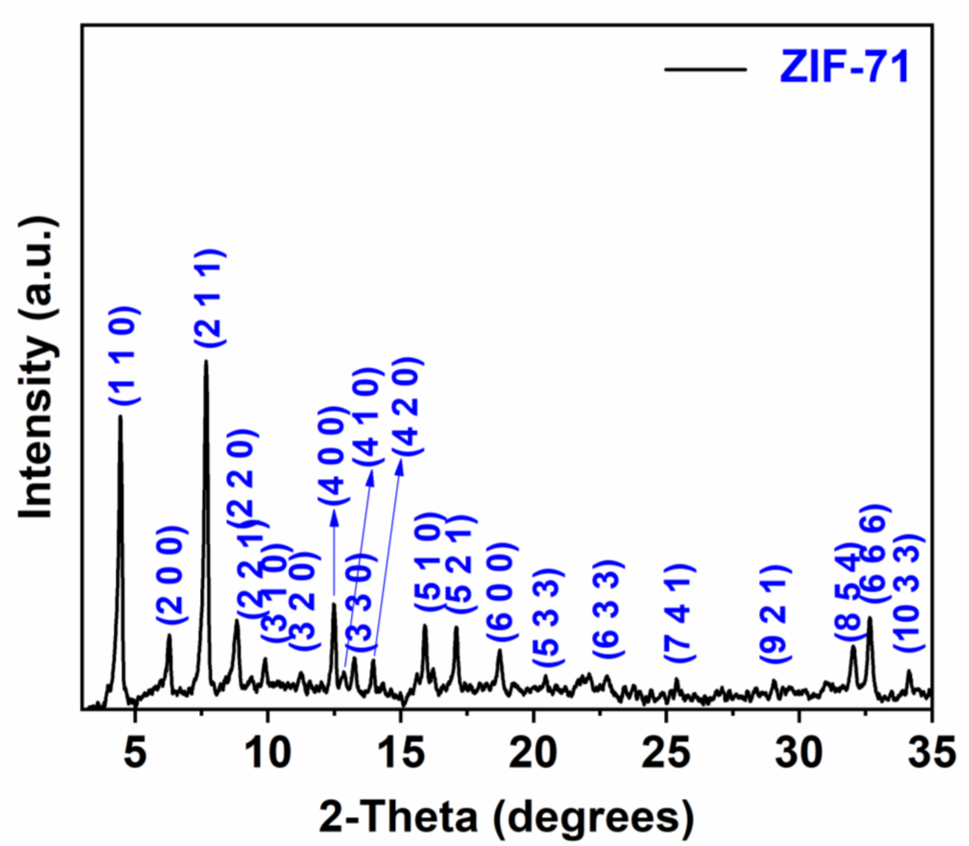

Figure S4. X-ray diffraction pattern of ZIF-71 nanoparticles.

Surface analysis using Arrhenius plot

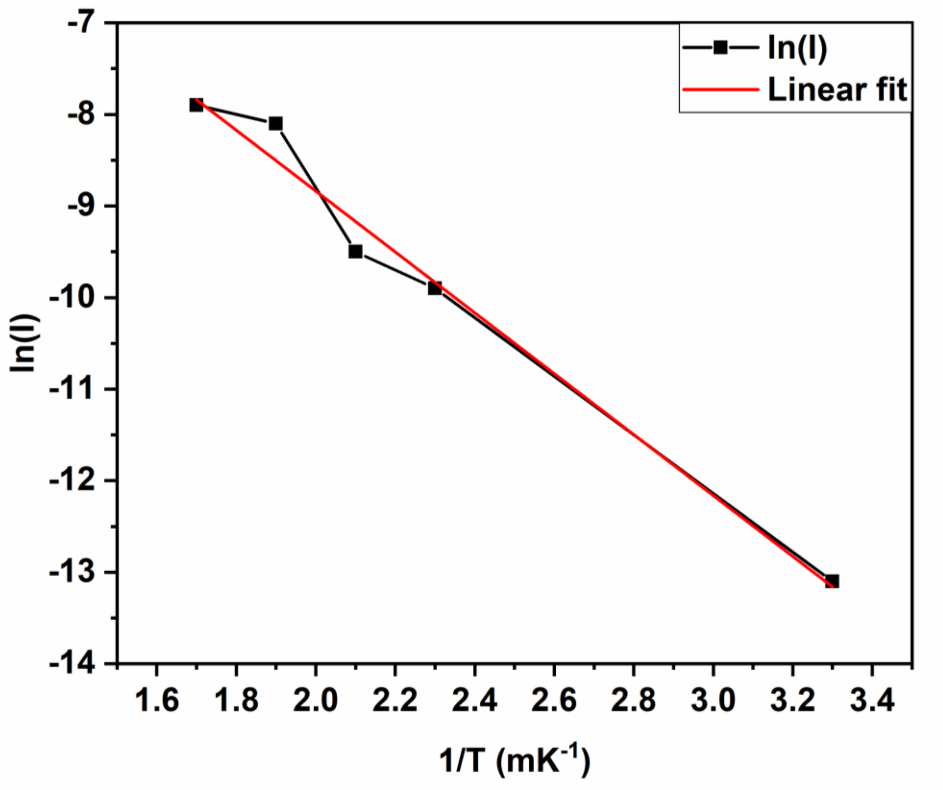

Figure S5. The Arrhenius plot of ln (I) vs 1/T for the calculation of activation energy.

Figure S5 presents an Arrhenius plot, where the natural logarithm of the measured current (ln(I), with I in μA) is plotted against the reciprocal of the operating temperature (1/T).

Energy band diagrams and Current-Voltage characteristics of metal semiconductor contacts.

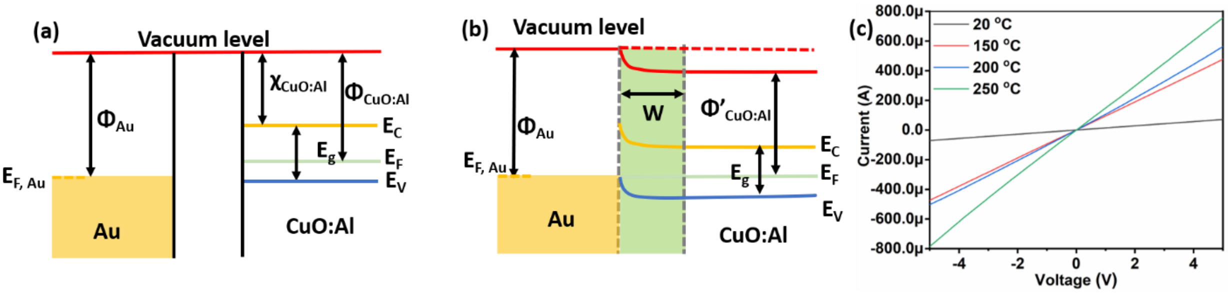

Figure S6. The energy band diagrams of Au and CuO (a) Before Ohmic contact. (b) After Ohmic contact. (c) Current-Voltage measurements of Au IDE (170 nm thick) contacts to p-type CuO:Al sample.

Study on gas sensing

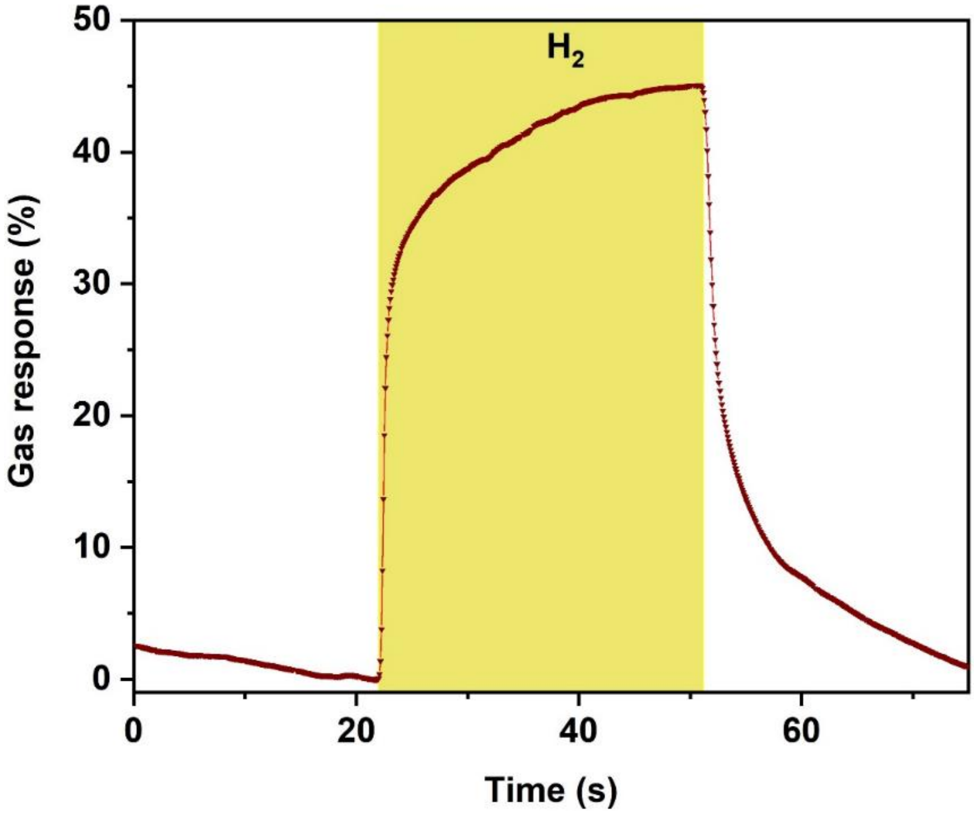

Figure S7. Dynamic response of the CuO:Al sensor to 100 ppm hydrogen at 250°C.

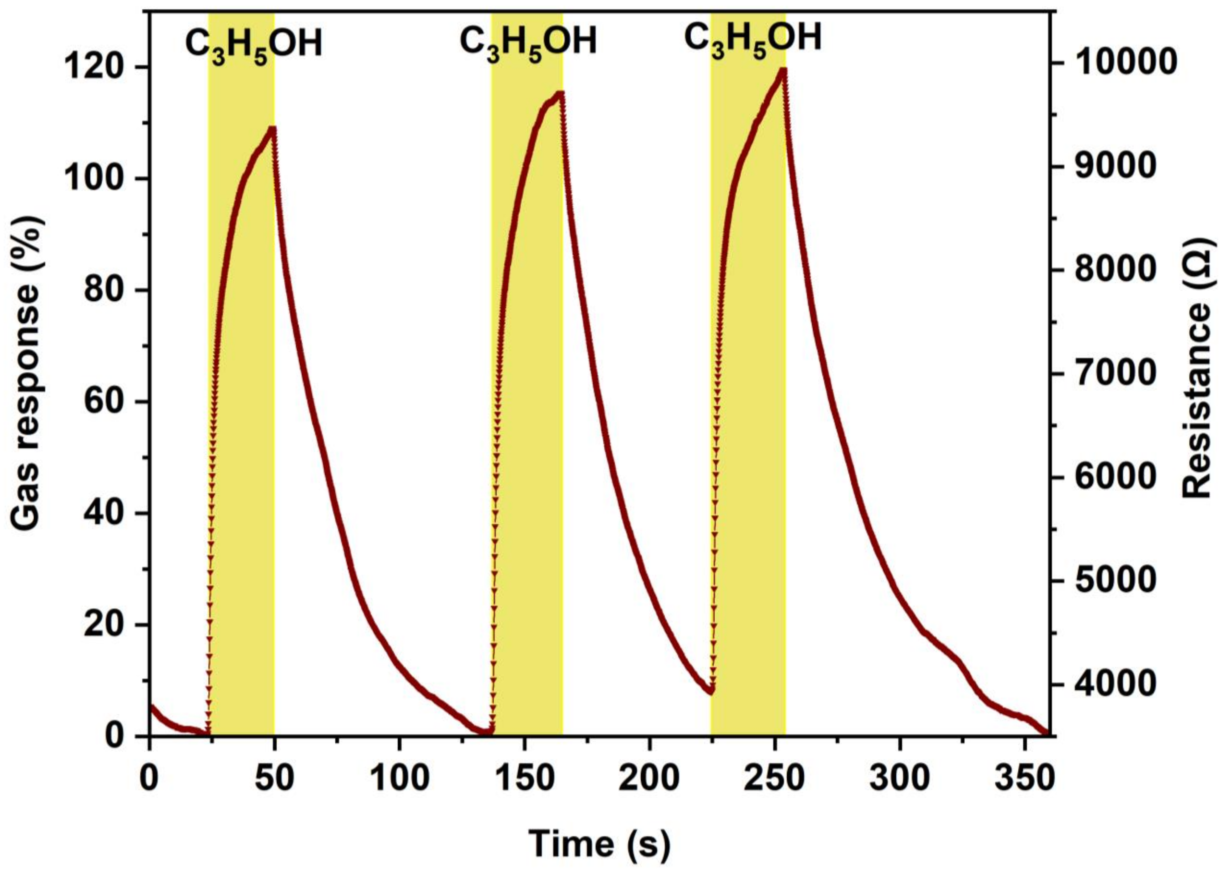

Figure S8. Dynamic response of the MOF ZIF-71-coated CuO:Al film-based hybrid sensor to 100 ppm acetone at 250°C at RH=10%, measured 21 days after the initial measurements.

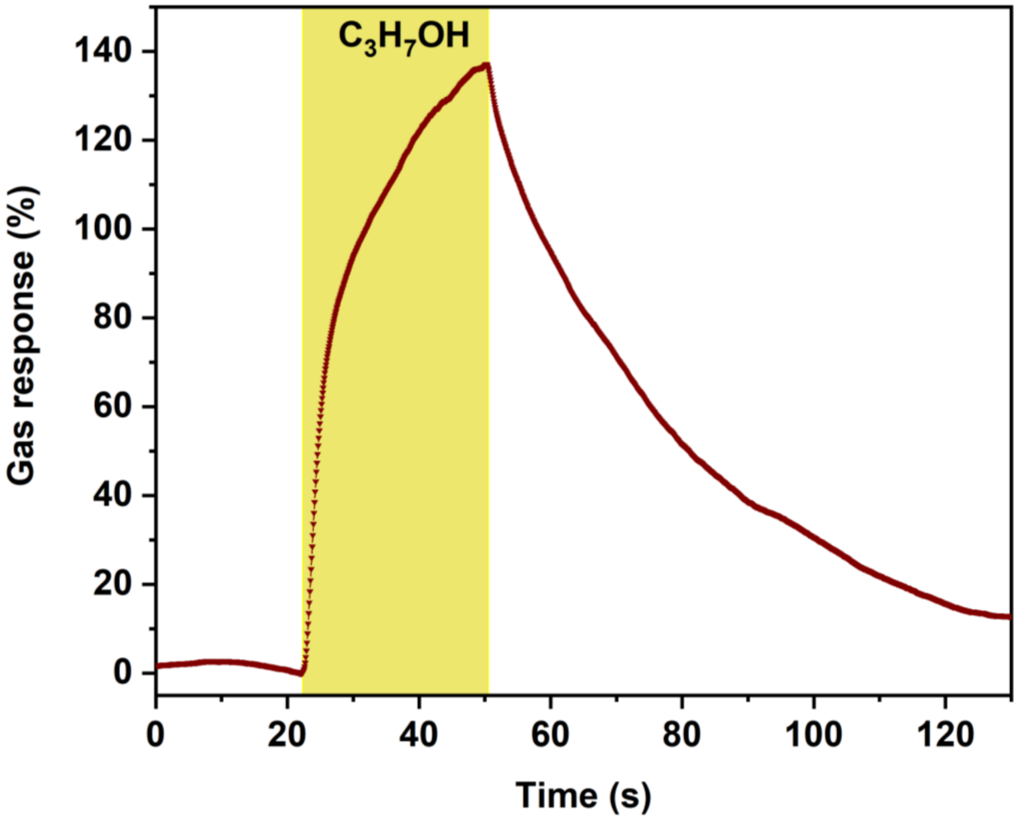

Figure S9. Dynamic response of the MOF ZIF-71-coated CuO:Al film-based hybrid sensor to 100 ppm 2-propanol at 250°C, measured 21 days after the initial measurements.

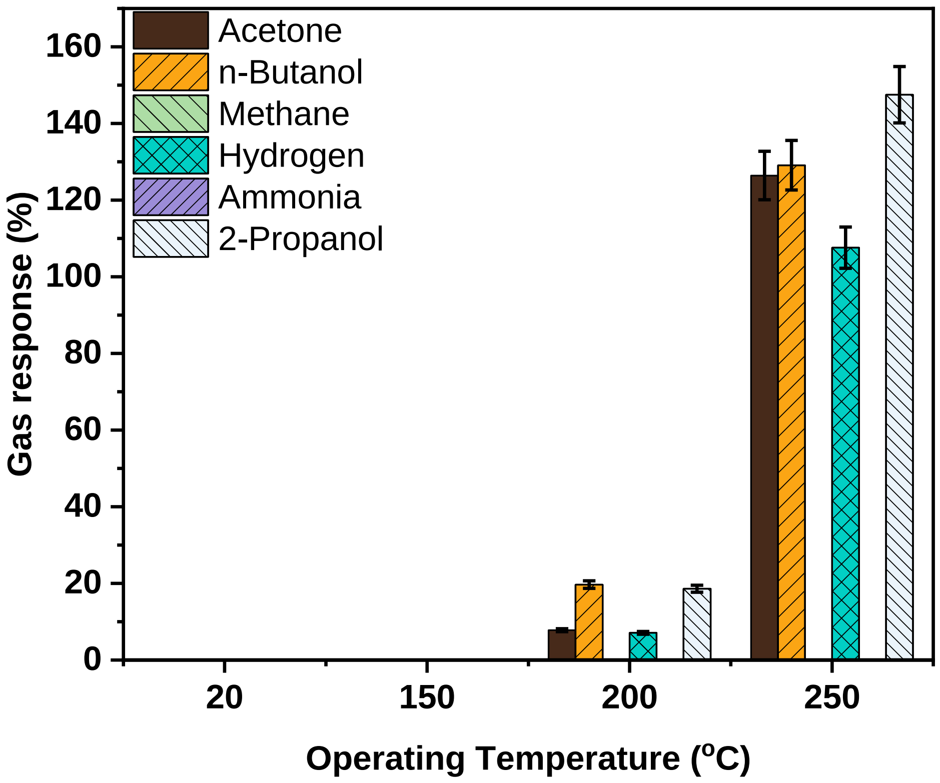

Figure S10. Gas sensing response to a series of gases with 100 ppm concentration at different operating temperatures for MOF ZIF-71-coated CuO:Al film-based hybrid sensor: after 42 days from the initial measurement at RH 10%.

Energy band diagrams for sensing mechanism

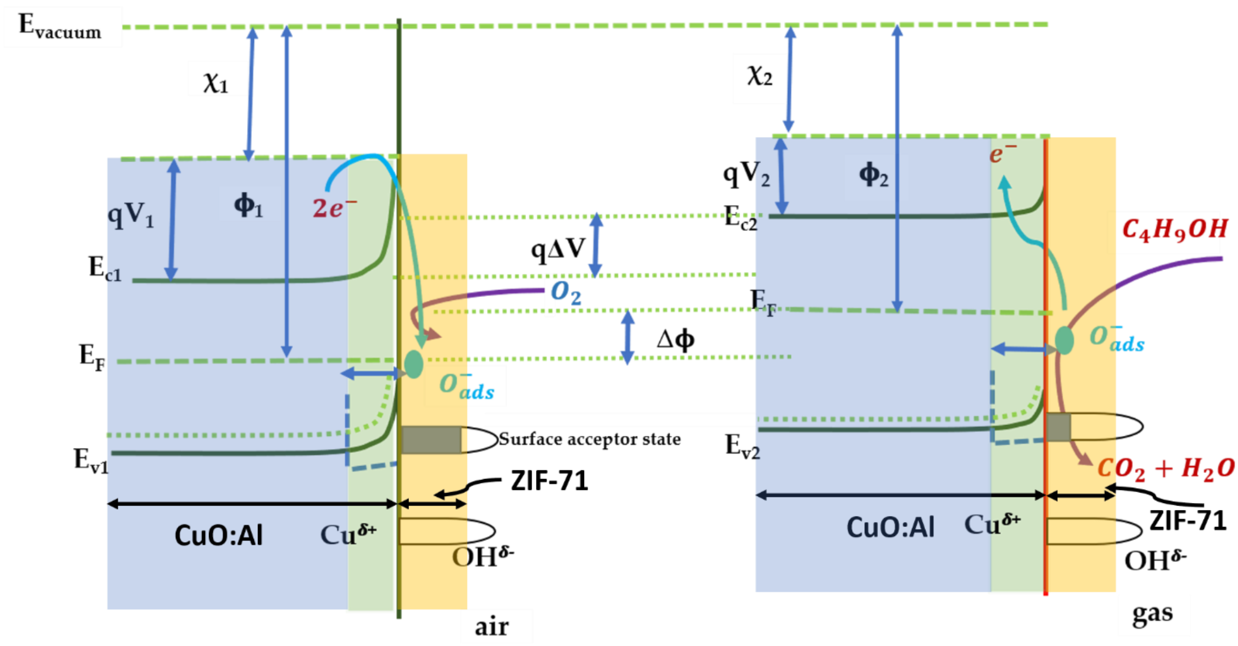

Figure S11. Gas sensing mechanism of the CuO:Al illustrated energy band diagrams towards n-butanol and other reducing gases; the absorption of oxygen species from ambient air; and in the presence of target gas (n-butanol).
